# Endocrinology at a Miniature Level: Pluripotent Stem-Cell-Derived Organoid Models of Hypothalamus–Pituitary Axes

**DOI:** 10.3390/biom16040558

**Published:** 2026-04-09

**Authors:** Berkehür Abaylı, Ulrieke Van Gestel, Hugo Vankelecom, Emma Laporte

**Affiliations:** Cluster of Stem Cell and Developmental Biology, KU Leuven, 3000 Leuven, Belgium; berkehuer.abayli@kuleuven.be (B.A.); ulrieke.vangestel@kuleuven.be (U.V.G.); hugo.vankelecom@kuleuven.be (H.V.)

**Keywords:** human embryonic stem cells, human-induced pluripotent stem cells, organoids, endocrinology, hypothalamus, pituitary, thyroid gland, adrenal gland, ovary, testis

## Abstract

Pluripotent stem cells (PSCs) have proven outstanding potential to revolutionize biomedical research. Specifically, their capacity to form 3D multicellular systems that recapitulate organ development and biology, known as organoids, has transformed basic and translational research. The groundbreaking technology is also being applied to the intricate hypothalamus–pituitary (HP) axes, including the target organs (such as gonads, thyroid and adrenal glands). These HP axes govern critical physiological processes, including reproduction, metabolism and stress. Here, we provide an overview of PSC-derived organoid models that are part of the HP axes, both as individual and multi-organ systems, and evaluate their culturing conditions, phenotypic characteristics, advantages, drawbacks and challenges, as well as their potential for disease modeling and therapeutic discovery. By offering this wide perspective, our review will also serve as a key resource for researchers navigating the evolving landscape of PSC-derived organoid technologies in endocrinology.

## 1. Introduction

**Figure 1:**
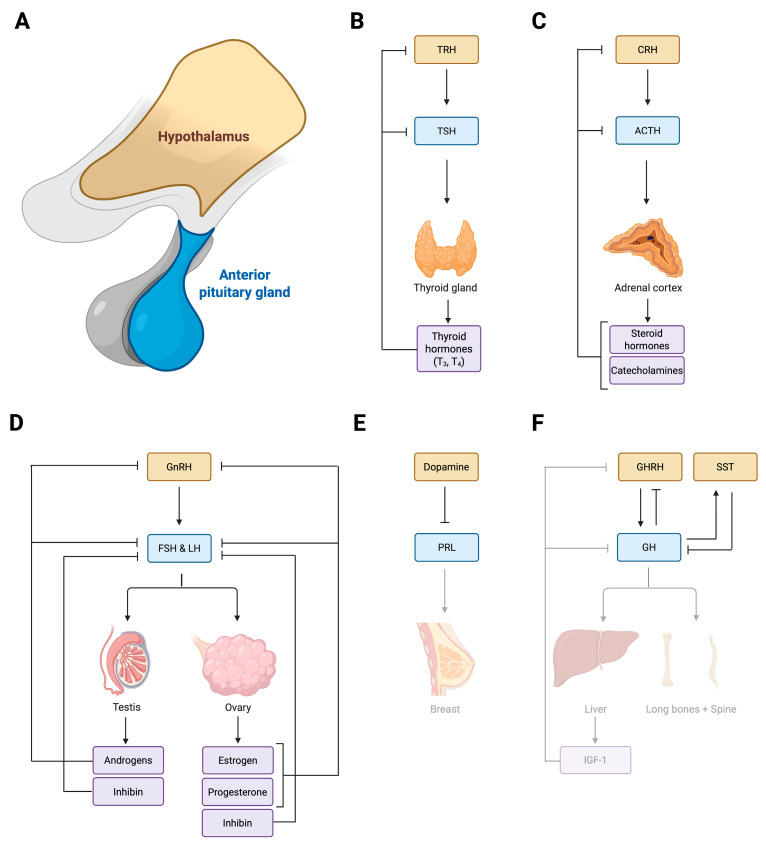
Human hypothalamic–pituitary endocrine axes (**A**–**F**). (TRH: thyrotropin-releasing hormone, TSH: thyroid-stimulating hormone, T3: triiodothyronine, T4: thyroxine, CRH: corticotropin-releasing hormone, ACTH: adrenocorticotropic hormone, GnRH: gonadotropin-releasing hormone, FSH: follicle-stimulating hormone, LH: luteinizing hormone, PRL: prolactin, GHRH: growth hormone-releasing hormone, GH: growth hormone, SST: somatostatin). Transparent signs and symbols indicate organs and mechanisms that are covered in this review. Created in BioRender. Vankelecom, H. (2026) https://BioRender.com/4kj30f1.

Serving as master regulators, the hypothalamus and pituitary gland (Figure 1A) coordinate the endocrine system [1]. Together they constitute the HP axis, wherein the hypothalamus interprets signals from the body, representing its physiological needs, and provides a response in the form of releasing hormones. These releasing hormones travel through the pituitary stalk to induce pituitary hormone secretion and regulate downstream target organs controlling metabolism, stress responses, reproduction, and growth [2,3]. Metabolism is coordinated through the release of thyrotropin-releasing hormone (TRH) from the paraventricular nucleus, prompting thyroid-stimulating hormone (TSH) production within thyrotropes, targeting the thyroid gland and inducing the release of thyroxine (T4) and triiodothyronine (T3) (Figure 1B) [4,5,6]. In addition, the same nucleus coordinates the regulation of stress response by secreting corticotropin-releasing hormone (CRH), thereby triggering adrenocorticotropic hormone (ACTH) production in corticotropes and inducing the release of steroid hormones and catecholamines in the adrenal gland (Figure 1C) [7,8]. Furthermore, the regulation of reproductive function is mediated through the production of gonadotropin-releasing hormone (GnRH) in the medial preoptic area, enhancing secretion of both luteinizing hormone (LH) and follicle-stimulating hormone (FSH) in gonadotropes, controlling folliculogenesis and oocyte maturation together with synthesis of androgens, estrogens and progesterone (PG) in the ovaries (Figure 1D) [9,10,11]. Meanwhile, in the testes, LH and FSH govern androgen production and spermatogenesis (Figure 1D) [12]. Additionally, lactation is governed by dopamine release from tuberoinfundibular dopamine neurons present in the arcuate nucleus of the hypothalamus, stimulating prolactin (PRL) secretion and promoting lactation (Figure 1E) [13]. Moreover, growth is primarily stimulated by the release of growth-hormone-releasing hormone (GHRH) from the arcuate nucleus of the hypothalamus [14], resulting in stimulation of growth hormone (GH) synthesis in somatotropes, driving hepatic IGF-1 production which, together with GH, initiates bone growth in the long bones and spine (Figure 1F) [15].

Even though in vivo models have played a pivotal role in studying systemic feedback loops and neuroendocrine interactions, these models are limited by inter-species differences and consequently their translational capacity towards humans [16]. Human PSCs (hPSCs) have emerged as a powerful tool for overcoming species-specific limitations and can be classified into two main categories: embryonic stem cells (ESCs) derived from the inner cell mass of blastocysts and induced pluripotent stem cells (iPSCs) generated by reprogramming fibroblasts [17]. Overall, PSCs are characterized by two core properties: self-renewal and potency. Self-renewal refers to the ability to expand while preserving an undifferentiated state, whereas potency refers to the capacity of differentiation into specialized cell types representing the three primary germ layers: ectoderm, mesoderm, and endoderm [18]. Importantly, PSCs secrete extracellular vesicles that act as paracrine modulators, delivering bioactive cargo to recipient cells and influencing their metabolism, survival, and differentiation [19]. PSCs enable the generation of two-dimensional (2D) in vitro models that circumvent inter-species differences. Nevertheless, 2D models fall short in recapitulating 3D cell–cell interactions, and multicellular complexity inherent to the native organs and glands [20]. To close this gap, researchers have established innovative 3D models known as organoids, which can be derived from hPSCs. These hPSC-derived organoid models are generated through processes that recapitulate key aspects of embryonic development and therefore are a promising tool to study organs and glands in a laboratory setting [21,22].

Remarkably, the integration of organoid models with organ-on-chip technology could, in the future, provide a platform to study interactions across HP–target organ axes. Organ-on-chip platforms comprise microfluidic devices designed to culture human cells under precisely controlled flow conditions. Through recreating dynamic perfusion, tailored microenvironments, and compartmentalized tissue organization, these systems provide a powerful framework for modeling complex inter-tissue communication and endocrine axis function [23].

In this review, we outline the current progress in HP axis modeling. We explore how existing models could be integrated and what their limitations are, and highlight potential future directions. We selected studies that developed organoid models of the HP–endocrine axes using human ESCs and/or human iPSC lines. Studies incorporating somatic cell types derived from adult human tissue were also included, provided that ESCs and/or iPSCs were used as a core component of the model. In addition, we aimed to include all relevant protocols, encompassing both peer-reviewed publications and preprints, to comprehensively summarize the characteristics and functionalities of existing models.

## 2. Hypothalamus Organoids

**Figure 2:**
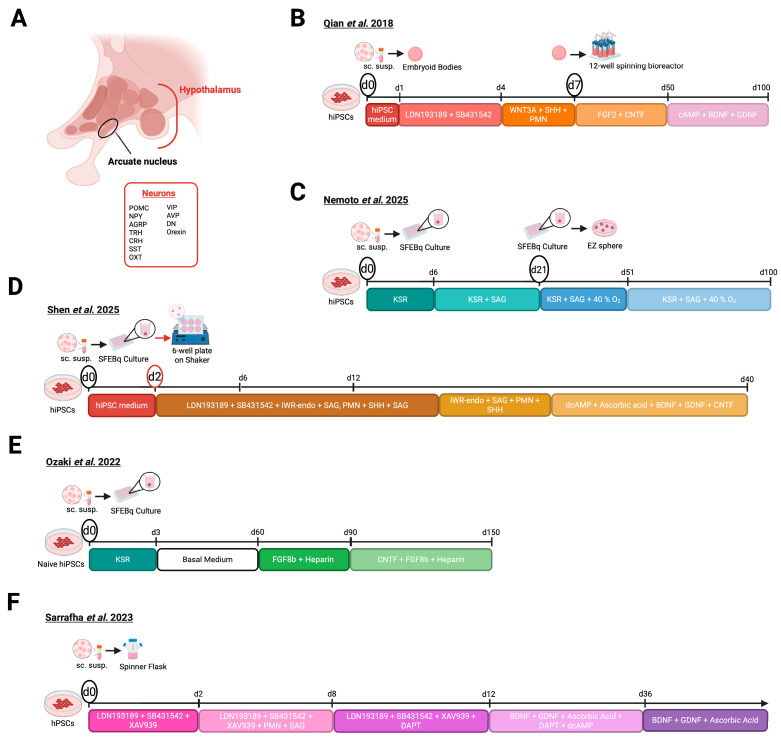
hESC/hiPSC-derived hypothalamus organoids. (**A**) Anatomy of the human hypothalamus. (**B**–**F**) hESC/hiPSC-based hypothalamic organoid generation methods developed by Qian et al. [27] (**B**), Nemoto et al. [28] (**C**), Shen et al. [29] (**D**), Ozaki et al. [30] (**E**), and Sarrafha et al. [31] (**F**). (hESC: human embryonic stem cell, hiPSCs: human-induced pluripotent stem cells, SHH: sonic hedgehog, PMN: purmorphamine, d: day, sc. susp.: single-cell suspension, SFEBq: three-dimensional aggregation culture, KSR: knock-out serum replacement). Created in BioRender. Vankelecom, H. (2026) https://BioRender.com/6nzmsfd.

The hypothalamus (Figure 2A) exhibits a structurally complex organization, encompassing four distinct regions: the supraoptic, tuberal, preoptic and mammillary region. Each region is subdivided into three zones: the periventricular, medial and lateral zone [24]. These zones accommodate 3D neural clusters called nuclei, and each nucleus is specialized to modulate distinct physiological functions [25]. Given the hypothalamus’s central role in regulating the endocrine system, there is a need for models that recapitulate its structure and interactions to deepen our understanding of its biology. A variety of PSC-derived models have been established to study this intricate system, which we list here.

In 2018, Qian et al. pioneered hypothalamic research by introducing the first protocol (Figure 2B) to generate hypothalamus organoids from hiPSCs [26]. The protocol starts with detaching hiPSC colonies, followed by seeding the cells into an ultra-low attachment plate to induce embryoid body (EB) formation. Pre-patterning of the hiPSCs to neuroectodermal fate was supported by the addition of the dual-SMAD inhibitors (SB431542 and LDN193189) and knock-out serum replacement (KSR). After 3 days, hypothalamic lineage was induced by treating the EBs with purmorphamine (PMN) and Sonic hedgehog (SHH) to activate the SHH pathway and WNT3A, and to stimulate the WNT pathway. On day 7, organoids were placed in a spinning bioreactor, KSR was omitted from the medium, whereas CNTF and FGF2 were incorporated to support neuronal development. From day 50 onward the medium was supplemented with cAMP, BDNF (to promote neuronal survival and differentiation) and GDNF (to aid formation of dopaminergic neurons), while CNTF and FGF2 were excluded (Figure 2B) [26].

Immunofluorescent (IF) staining revealed that, from day 8, organoids express markers present during hypothalamic development, including *NKX2.1*, *NKX2.2*, RAX1, NESTIN, SOX2 and FOXA2, confirming hypothalamic identity. On day 40, expression of peptidergic neuronal markers including pro-opiomelanocortin (POMC), neuropeptide Y (NPY), vasoactive intestinal peptide (VIP), and oxytocin (OXT) was detected in 15–25% of cells depending on the cell line used. Additionally, the presence of the key transcription factor (TF), OTP, suggests progressive maturation [32]. Altogether, Qian et al. successfully generated hypothalamic organoids as evidenced by the expression of characteristic markers (Table 1) [27]. However, detailed characterization of the hormone-producing cell types, particularly those mediating pituitary communication, was absent. Additional assays are needed to confirm if the generated neurons within the organoid model reach functional maturity.

Whereas Qian et al. sought to generate a broad hypothalamic organoid model, Huang et al. (2021) [33] focused on establishing an organoid model of the arcuate nucleus to investigate Prader–Willi syndrome (PWS), a disease caused by the loss of paternal gene expression on chromosome 15, characterized by hypothalamic dysfunction [52]. To generate arcuate nucleus organoids (ARCOs), hiPSC colonies were detached and plated in ultra-low attachment plates harboring induction medium for neuroectoderm specification through dual-SMAD inhibition. Additionally, IWR-1-endo was supplemented to inhibit WNT signaling, and SAG, PMN and SHH to induce hypothalamic patterning. From day 3 onwards, organoids were maintained on an orbital shaker and from day 6, LDN193189 and A83-01 were excluded from the medium. Starting on day 12, organoids were cultured in differentiation medium consisting of astrocyte conditioned medium and trophic factors, i.e., BDNF and GDNF, to promote hypothalamic neuron maturation [33,53].

Similar to Qian et al. [26], hypothalamic progenitor markers *NKX2.1*, *NKX2.2* and RAX were detected during the early stages of differentiation. Additionally, organoids revealed an absence of PAX6, a forebrain neural progenitor cell (NPC) marker, suggesting ventral patterning. Moreover, on day 40, Huang et al. confirmed robust expression of arcuate nucleus markers including OTP and POMC, also observed by Qian et al., as well as DLX1 and TBX3 [26,33]. Additionally, diverse hypothalamic neuron markers such as NPY (also observed in Qian et al.), SST, ISL1, and PV were detected. Furthermore, Huang et al. performed single-nucleus RNA sequencing (snRNA-seq) of human neonatal hypothalamus samples and ARCOs to assess their resemblance [33]. From this analysis, Huang et al. concluded that the ARCOs exhibit comparable cell type diversity and molecular signatures [33]. A total of 14 broad clusters were identified from ARCO samples, encompassing various cell types such as NPCs (*NESTIN*+, *ASCL1*+, *SOX2*+), intermediate progenitor cells (IPCs; *OTP*+, *DLX1/2*+, *CRABP1*+), and neurons (*MAP2*+, *RBDOX3*+/*NEUN*+). Notably, specific populations showed prominent levels of arcuate nucleus markers, including *POMC*, *OTP*, *DLX1*, and *TBX3* [33].

Next, PWS hiPSC lines were established and used to develop PWS ARCOs. Huang et al. conducted bulk RNA-seq to evaluate transcriptional changes between PWS and control group ARCOs to demonstrate that PWS organoids retain disease-relevant and patient-specific gene signatures. As impaired leptin signaling is a hallmark of PWS, Huang et al. investigated this pathway in both the control and PWS ARCOs after leptin treatment. PWS ARCOs have decreased levels of JAK2 and STAT3 and lower MSH secretion, both suggesting impaired leptin signaling and substantiating the model’s recapitulative strength. Altogether, Huang et al. developed a novel hypothalamus organoid model (Table 1) that could be harnessed in future studies to investigate arcuate nucleus development and uncover mechanisms of pathobiology [33].

Excitingly, Nemoto et al. (2025) [28] used CRISPR-based epigenetic editing to demethylate a set of epigenetically silenced genes on the maternal allele in patient hiPSCs, a defining feature of PWS [54]. These edited cells were subsequently differentiated into hypothalamus organoids (Figure 2C) [28]. hiPSCs were dissociated and plated into an ultra-low attachment plate containing differentiation medium supplemented with KSR to initiate serum-free culturing of EB-like aggregates with quick reaggregation (SFEBq). Between day 6 and 15, SAG was supplemented to activate the SHH pathway, and from day 21, the organoids were cultured under hyperoxic conditions (40% O_2_). Starting from day 51, the concentration of KSR was increased from 10% to 20% (Figure 2C).

IF staining on day 30 revealed the presence of hypothalamic markers RAX, *NKX2.1* and OTP. Furthermore, between day 63–66, Nemoto et al. performed single-cell (sc) RNA-seq and compared the obtained data with the dataset reported by Huang et al., hereby further substantiating the formation of hypothalamus organoids. Moreover, PWS hiPSC-derived hypothalamus organoids showed reduced expression of key genes *SNORD116*, *IPW* and *MAGEL2*, and diminished spontaneous neuronal activity, consistent with patient pathology. Notably, demethylation of the PWS imprinting control region restored gene expression and activity to levels comparable with healthy organoids. In addition, post mortem hypothalamus samples from PWS patients revealed an upregulation of ribosome-related genes, while genes related to synaptic activity were downregulated compared to healthy hypothalamus samples [55]. This pattern is reversed in the hypothalamus organoids generated from epigenetically edited hiPSCs. Overall, Nemoto et al. provide a novel organoid model (Table 1) to study PWS, advancing mechanistic insight, therapeutic prospects, and opportunities for epigenome editing in disease research [28].

Recently, Shen et al. (2025) refined the protocol by Huang et al., resulting in less organoid-to-organoid variability and reduced cell death (Figure 2D) [29]. Additionally, this method was optimized for feeder-free (Ff) hiPSCs. Furthermore, the established ARCOs were utilized to investigate how m^6^A deficiency affects hypothalamic neurogenesis of feeding-related neurons that control hunger and satiety (NPY and POMC neurons, respectively). Different from Huang et al., hiPSCs were detached and plated into ultra-low attachment plates harboring stem cell medium for 24 h to induce EB aggregation. From day 2 onwards, EBs were transferred into a new plate holding induction medium and placed on an orbital shaker. Compared to Huang et al., this medium additionally included KSR and heparin. As of day 6, dual-SMAD inhibition was lifted and KSR and heparin were excluded from the medium. From day 12 onward, organoids were cultured in differentiation medium containing CNTF, BDNF, GDNF, B27, dcAMP and ascorbic acid to promote hypothalamic neuron maturation (Figure 2D) [29]. As anticipated, by day 12, IF staining demonstrated the detection of hypothalamic NPC markers, *NKX2.1* and MASH1. Furthermore, transcriptional analysis revealed a substantially increased expression of hypothalamic NPC markers *NKX2.1*, *RAX* and *ASCL1* on day 13, in agreement with previous findings from Huang et al. Additionally, expression of hypothalamic neuronal markers *MAP2*, *NPY*, *MC4R*, and *TH* showed a gradual increase between day 20–40, as seen in Huang et al. IF staining on day 30 illustrated the presence of neuronal markers POMC, αMSH, and OTP, as previously reported in Huang et al., as well as TH. To assess the effect of m^6^A deficiency on feeding-related neurons, the generated ARCOs were transfected on day 11 with lentiviruses-expressing short hairpin RNA against mediators of m^6^A signaling, specifically *METTL14* and *YTHDC1*. This led to a decreased expression of POMC and NPY, implying a role for m^6^A signaling in the generation of human feeding-related neurons. Overall, Shen et al. presents an enhanced version of the protocol of Huang et al., achieving reduced organoid-to-organoid variability and less cell death, while preserving neuronal diversity (Table 1) [29]. Additionally, they provide a novel tool to study m^6^A deficiency in the arcuate nucleus.

In 2022, Ozaki et al. applied an innovative strategy involving early withdrawal of exogenous signals to generate rostral hypothalamus organoids containing AVP+ neurons. The resulting model was employed to investigate familial neurohypophyseal diabetes insipidus (FNDI), a disease defined by degeneration of AVP+ neurons, resulting in dysregulated water homeostasis (Figure 2E) [30]. Previously, Mitsumoto et al. (2019) established a protocol to develop AVP+ neurons from mouse iPSCs, where a similar approach controlled positional cues to induce rostral patterning [56]. Following the conversion of hiPSCs to a naïve-like state (essential for cell survival and consequently successful differentiation), the naïve hiPSCs were dissociated to single cells [57,58,59,60] and transferred into a plate containing gfCDM, KSR and Y-27632 (inhibitor of p160ROCK) [61]. KSR was withdrawn on day 6 and Y-27632 on day 9. After 30 days, aggregates were transferred to a dish for suspension culturing. Starting from day 60, heparin and FGF8b were added to the medium to induce AVP+ neuron formation. After day 90, CNTF was added to the medium to further support neuronal maturation (Figure 2E) [30]. On day 30, IF staining revealed the presence of PAX6, a forebrain NPC marker, alongside the absence of the ventral hypothalamic marker *NKX2.1*, indicating the presence of dorsal hypothalamic progenitors and confirming correct patterning. The presence of OTP, expressed as well in Qian et al. and Huang et al., and BRN2, identified AVP precursor neurons. Additionally, on day 150, successful differentiation into AVP neurons was confirmed, as evidenced by the co-expression of AVP, NPII, and copeptin. Simultaneously, the presence of other hypothalamic markers was confirmed, such as CRH, AgRP, TRH, orexin, and NPY. To evaluate this model as a platform for studying FNDI, differentiation was performed using the control and patient-derived hiPSCs. The presence of mutant NPII, a hallmark of FNDI, was only found in patient-derived organoids [62]. Taken together, Ozaki et al. (2022) established a protocol for the induction of rostral hypothalamic organoids (Table 1) containing AVP+ neurons by application of a novel strategy involving the minimization of exogeneous signals [30]. Additionally, the presence of mutant NPII suggests that this model may serve as a valuable tool to study FNDI. However, functional tests regarding the activity of the neuronal population will be required in future studies to further validate the model’s applicability towards FNDI research.

Notably, 1 year later, Miwata et al. (2023) [34] applied fluorescence-activated cell sorting (FACS) to isolate hypothalamic cell types from the HP organoid model, previously established by Ozone et al. (2016) (described extensively below in the Section 3) [35], enabling the generation of neurospheres [34]. Characterization of the HP organoid model through IF staining on day 30 confirmed the expression of hypothalamic progenitor cell markers RAX, *NKX2.1* and PAX6. Furthermore, by day 90, expression of POMC, NPY, AGRP, and NR5A1, mature neuronal markers of the arcuate and ventromedial nuclei of the hypothalamus, was evident. Altogether, this suggests that the hypothalamic-like tissue within the HP organoid model reached maturation by day 90. Additionally, a distinct population of cells co-expressing RAX, SOX2, VIMENTIN, and NESTIN was observed, suggesting the presence of stem-cell-like niches within the hypothalamic compartment. To form neurospheres, RAX+ cells were isolated from the HP organoids and seeded into ultra-low attachment plates. The sorted RAX+ population successfully formed neurospheres that demonstrate self-renewal capacity, multipotency and capacity to differentiate into hypothalamic neurons such as NPY- and POMC-neurons both in vitro and after xenografting into severe combined immunodeficient (SCID) mice. Overall, Miwata et al. introduced a novel HP organoid-derived neurosphere model (Table 1), providing a powerful platform to study hypothalamic neural stem-cell-like cells and marking a major advance in regenerative strategies aimed to restore hypothalamic function [34].

In the same year, Sarrafha et al. (2023) introduced a protocol to generate hypothalamic organoids enriched with TH+ neurons (Figure 2F) [31]. The protocol started with the dissociation of hiPSCs or hESCs, hereafter seeded as single cells into a spinner flask to initiate hypothalamic patterning. On day 0–1, the medium was supplemented with LDN193189, SB431542 and XAV939 to promote floor plate formation through dual-SMAD inhibition and to induce forebrain patterning. PMN and SAG were added between day 2–7 to initiate ventralization of the developing NPCs. DAPT was incorporated into the medium between day 8–11. From day 12 onwards, the base medium was replaced by terminal differentiation medium consisting of BDNF, GDNF, ascorbic acid, dcAMP and DAPT to induce commitment towards dopaminergic lineage and support neuronal maturation. On day 22, BDNF and GDNF concentrations were halved to avoid masking dopaminergic neuron-related pathology. Starting from day 36, DAPT and dcAMP were removed for the same reason (Figure 2F) [31]. On day 15, 97% of cells were *NKX2.1*+, indicating that the organoids adopted ventral forebrain identity. In contrast, OTP was present in minimal amounts compared to other protocols [27,29,33], indicating a limited presence of OXT+, AVP+, CRH+, and SST+ neurons in the organoids [32,63]. Furthermore, characterization of the organoids on day 80 confirmed dopaminergic neuron fate by the presence of TH+ cells. Moreover, POMC expression was detected, consistent with prior observations across all hypothalamic models presented in this review. Additionally, TRH-expressing neurons were identified, uniquely reported before by Ozaki et al. To assess the activity of the neuronal population, extracellular recordings of spontaneous electrical activity were conducted using multi-electrode arrays. Measurements performed on day 101 and 108 revealed periodic bursts, indicating that the protocol yields electrophysiological active neurons upon maturation. To further characterize the organoid model, scRNA-seq was employed, revealing that neuronal cell types (*DCX*+) comprised 88.7% of the total cell population. This neuronal cluster could be divided into 17 subtypes: 31.5% was classified as unknown, 26.4% belonged to neuro-endocrine-like cells (*RCN3*+), 11.9% DN-neurons (*TH*+), 8.2% SF1-neurons (*NR5A1*+, *SLC17A6*+), 1.1% TRH-neurons (*TH*+, *SLC17A6*+, *TAC1*+, *TRH*+), 0.7% OXT-neurons (*OXT*+), and 0.3% POMC-neurons (*TH*+, *TAC1*+, *HCRT*+, *TRH*+, *POMC*+). The main other cell types present in the organoid model include astrocytes (*GFAP*+), radial glial cells (*TOP2A*+), and IPCs (*ASCL1*+). Altogether, Sarrafha et al. developed a hypothalamic organoid model enriched in neuronal cell types (Table 1) that display electrophysiological activity. Still, the identity of the major unidentified neuronal cluster remains to be determined [31].

## 3. Pituitary Organoids

**Figure 3:**
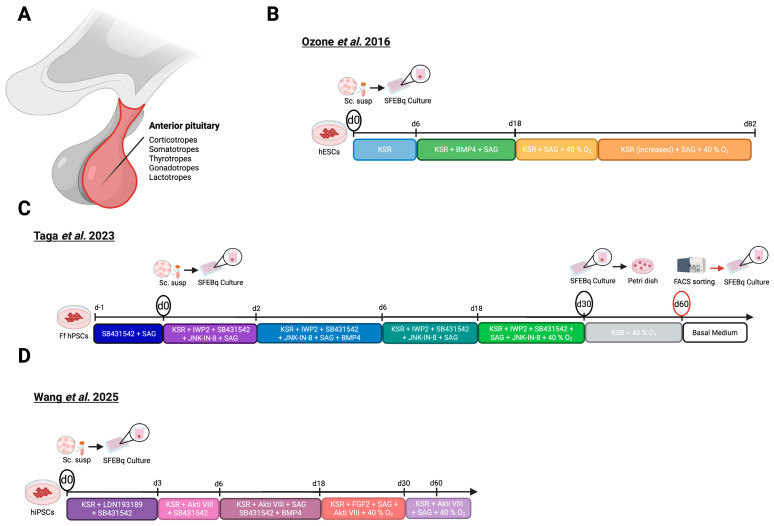
hESC/hiPSC-derived HP organoids. (**A**) Anatomy of the human pituitary. (**B**–**D**) hESC/hiPSC-based HP organoid generation methods developed by Ozone et al. [35] (**B**), Taga et al. [38] (**C**), and Wang et al. [39] (**D**). (hESC: human embryonic stem cell, hiPSCs: human-induced pluripotent stem cells, d: day, sc. susp.: single-cell suspension, SFEBq: three-dimensional aggregation culture, KSR: knock-out serum replacement, Ff hiPSCs: feeder-free hiPSCs, FACS: Fluorescence-activated cell sorting). Created in BioRender. Vankelecom, H. (2026) https://BioRender.com/ijen4ib.

Even though the hypothalamus initiates endocrine regulation, the pituitary gland (Figure 3A), often referred to as the master gland, plays a pivotal role in implementing its signals. In humans the pituitary comprises two lobes: the anterior lobe, encompassing two distinct regions, the *pars tuberalis* and *pars distalis*, and the posterior lobe [1]. The *pars tuberalis* harbors hypophyseal portal vessels and epithelial cells, whereas the *pars distalis* serves as the principal site for hormone production and contains five hormone-producing cell types, including corticotropes, somatotropes, lactotropes, thyrotropes, and gonadotropes [10,64]. Recognized for its pivotal role in coordinating endocrine regulation, the pituitary gland has become a prominent target for in vitro modeling. Currently, protocols for generating hPSC-derived pituitary organoids rely on hybrid organoids (known as HP organoids) that contain both the hypothalamus and pituitary, as the hypothalamus plays a crucial role in both the development and maturation of the pituitary gland in vivo [36,65].

The journey of HP organoid development commenced in 2016, when Ozone et al. aimed to generate HP organoids through recapitulation of murine pituitary embryonic development using hESCs (Figure 3B) [35]. The protocol is initiated by dissociating hESCs and seeding the cells into ultra-low attachment plates for SFEBq culturing. Under these conditions, the cells rapidly reaggregate into uniform 3D spheroids, which subsequently undergo directed differentiation [66]. Through SFEBq culturing, combined with an optimized medium and a high cell seeding density, Ozone et al. aimed to mimic the emergence of the pituitary primordium from the oral ectoderm induced by ventral hypothalamic neuroepithelium. At organoid induction, the medium, composed of gfCDM [61], was supplemented with KSR. On day 6, SHH and BMP signaling, essential pathways for early pituitary development, were activated by addition of SAG and BMP4, respectively. SAG addition led to the formation of ventral hypothalamus-like tissue as evidenced by the presence of *NKX2.1*+ and RAX+ cells on day 24. BMP4 treatment led to the formation of non-neural ectoderm, surrounding the ventral-hypothalamic-like tissue, characterized by the expression of CK and ECAD. Excitingly, the layer of non-neural ectoderm positioned at the periphery of organoids exhibited expression of PITX1, an early marker of pituitary and oral ectodermal identity. From day 18, BMP4 was gradually removed from the medium and aggregates were cultured under hyperoxic (40% O_2_) conditions to promote GH secretion. Notably, activation of FGF signaling between day 15–27 was followed by a prominent increase in vesicle-like structures, expressing early pituitary markers, PITX1 and LHX3, within the CK+ oral ectoderm, resembling Rathke’s pouch (a structure that gives rise to the anterior lobe of the pituitary gland during development). After 27 days, the organoids were transferred to an ultra-low attachment dish for suspension culturing. KSR levels were increased on day 27 and further elevated on day 50 (Figure 3B) [35]. Pituitary-hormone-producing cell types started to emerge around day 67, starting with the corticotropes, accounting for 12.1% of PITX1+ cells.

To assess HP organoid functionality, stimulatory or inhibitory compounds were applied to the medium, CRH or glucocorticoids, respectively, and ACTH secretion was measured. After administration of CRH, ACTH secretion increased while a decreased secretion occurred after glucocorticoid supplementation, indicating that the present corticotrope population can respond to both positive and negative hormonal feedback. Furthermore, IF staining revealed the presence of lactotropes (PRL+) and thyrotropes (TSH+), though they represented less than 2% and 1% of PITX1+ cells, respectively. Dexamethasone (DX) supplementation was used to drive somatotrope differentiation between day 72–84, resulting in 8.9% of PITX1+ cells differentiating to somatotropes on day 85. Somatotrope regulatory capacity was assessed using GHRH as a positive stimulus and somatostatin as an inhibitor. Somatotropes, like corticotropes, showed bidirectional feedback responses [35]. To promote gonadotrope specification, DAPT was added to the medium between day 72–82, which in an earlier study was shown to induce GATA2 precursor lineages including gonadotropes, resulting in a marked increase in LH+ and FSH+ cells [67].

To evaluate the model’s capacity to functionally substitute the pituitary within the hypothalamic–pituitary–adrenal (HPA) axis, kidney subcapsular transplantation was performed in hypophysectomized SCID mice. Subsequent measurements of basal ACTH levels showed increased secretion in transplanted mice compared to sham controls; however, these values remained below those observed in non-hypophysectomized animals, indicating a partial rescue. A comparable pattern was observed for basal corticosterone, with increased levels in transplanted mice relative to sham controls; nevertheless, with lower levels than in non-hypophysectomized animals. Additionally, functional CRH responsiveness was validated through CRH-loading tests at day 10 and 86 post-transplantation, eliciting significant ACTH increases at both timepoints. Transplantation improved behavior and survival relative to sham controls, albeit without reaching physiological levels. Altogether, this study provides a major advance in pituitary research, establishing an organoid model that expresses multiple hormones (Table 1) and responds to hormonal feedback [35]. However, the pituitary compartment remains largely composed of corticotropes and somatotropes. Additionally, functional responsiveness of other hormone-producing lineages remains untested, and a thorough characterization of the hypothalamic compartment is needed to fully assess the model’s capacity to recapitulate the HP axes.

Expanding on the foundational research by Ozone et al., Kasai et al. (2020) sought to adapt the protocol for hiPSCs through optimizing *LHX3* expression on day 40, given its importance in pituitary development [36]. Optimization of the protocol involved increasing the seeding density, changing the time of addition and concentration of KSR, and raising the BMP4 level. On day 30, the periphery of the HP organoid exhibited CK expression, indicative of oral-ectoderm-like tissue, while the interior displayed RAX expression, consistent with hypothalamic-precursor-like identity. From day 40, the oral-ectoderm-like compartment situated at the outermost region displayed LHX3, PITX1, and ECAD expression, reflecting early pituitary development. Additionally, RAX+ and *NKX2.1*+ ventral-hypothalamus-like cells were localized to the interior of the organoids, adjacent to the oral-ectoderm-like tissue. Altogether, these observations are in line with the results published by Ozone et al. and indicative of HP organoid formation. Corticotropes were the earliest hormone-secreting pituitary cell type detected, appearing by day 60. Nevertheless, Kasai et al. primarily focused on establishing a hiPSC-derived HP model, characterizing the hypothalamic compartment, and evaluating inter-compartmental communication between CRH+ neurons and corticotropes, rather than exploring the pituitary compartment within the organoids. Therefore, IF or gene expression data of pituitary hormone producing lineages were not present, except for corticotropes. IF staining performed on day 200 revealed the presence of distinct neuronal populations in the hypothalamic compartment, such as CRH+, AVP+, TRH+, AGRP+, NPY+, POMC+, MCH+, OXT+ and GHRH+ neurons [36].

Upon CRH stimulation, ACTH secretion increased, while DX reduced its secretion, demonstrating functional feedback control in HP organoids, aligning with the results previously described by Ozone et al. To investigate potential inter-compartmental communication, a low-glucose test was performed. Hypothalamic CRH-producing neurons respond to low-glucose concentrations by secreting CRH [68]. Indeed, ACTH secretion from the HP organoids increased in low-glucose medium. Treatment with CRH receptor 1 inhibitors, antalarmin or NBI 27914, before applying low-glucose conditions, diminishes this response, providing additional support to the hypothesis that functional communication occurs between both compartments. Altogether, Kasai et al. extended the scope of HP organoid differentiation to hiPSCs, and demonstrated the hormonal competence of the pituitary compartment as well as intercompartmental communication. Although characterization of pituitary cell types was limited (Table 1), IF staining revealed a diverse population of hypothalamic hormone-producing neurons [36]. Furthermore, the study focused exclusively on CRH-corticotrope signaling, leaving other HP axes to be explored.

While Kasai et al. focused on corticotrope hormonal output, Miyake et al. (2022) extended the study to lactotropes by assessing PRL secretion in HP organoids, which were generated using the same protocol [36,37]. From day 81 onward, PRL+ cells were observed, with their numbers and PRL secretion levels steadily rising over time. Furthermore, POU1F1 expression was noted from day 93 onwards, a TF crucial for lactotrope and somatotrope development [69]. Additionally, GATA2 expression was detected, indicating that this model may also be suitable for investigating gonadotropes or thyrotropes [70]. To study PRL-related feedback responses, stimulatory (Prolactin-Releasing Peptide 31 (PrRP31), VIP, TRH) or inhibitory (bromocriptine, a dopamine receptor agonist) factors were applied to the HP organoid cultures. The results demonstrated that PRL secretion was suppressed by bromocriptine and enhanced by PrRP31, VIP, or TRH, indicating correct responsiveness to inhibitory and stimulatory regulators of PRL production. Notably, Miyake et al. observed TH+ DN-neurons in the hypothalamic compartment connecting to PRL+ lactotropes. Overall, Miyake et al. showed that HP organoids generated using the Kasai et al. protocol contain lactotropes (Table 1) that respond to feedback [37]. However, functional assays are needed to determine whether dopaminergic neuron activity truly regulates PRL secretion, as connectivity does not imply functionality.

Whereas earlier protocols required feeder cells, Taga et al. (2023) established the first hPSC-derived pituitary organoid model using Ff hESCs and hiPSCs (Figure 3C) [38]. Furthermore, FACS-based isolation of the pituitary compartment excluded the hypothalamic region, yielding the first standalone hPSC-derived pituitary organoid model. Prior to differentiation, Ff hPSCs require preconditioning to facilitate self-organization and aggregate growth, as previously described by Kuwahara et al. [71]. Subsequently, hPSCs were dissociated into single cells and reaggregated in ultra-low attachment plates containing gfCDM [61] supplemented with JNK-IB-8, an inhibitor of the MAPK pathway, together with KSR, to promote formation of ectodermal-epithelium. Additionally, the medium contained IWP2 to block both canonical and non-canonical WNT pathways alongside SAG and SB431542 to induce placode formation [72]. Furthermore, BMP4 was applied on day 2, earlier than compared to previous protocols, alongside an increased concentration of SAG, to enhance placode induction. From day 6, concentrations of IWP2, SB431542, and SAG were maintained, whereas the BMP4 concentration was diluted stepwise. On day 30, the organoids were cultured together in one dish and maintained in gfCDM [61] supplemented with KSR without IWP2, SB431542, and SAG. From day 51, KSR concentration was increased (Figure 3C) [38].

Pituitary differentiation was monitored through *PITX1*, *LHX3*, and *POMC* expression, confirming appropriate progression of lineage specification. Additionally, hypothalamic differentiation was assessed through *RAX* and *NKX2.1* expression, indicating early-stage development of the ventral hypothalamus. IF staining on day 29 revealed the presence of LHX3+ cells localized to the periphery of the organoid, while RAX+/*NKX2.1*+ ventral hypothalamus neuroepithelium was detected in the interior, consistent with observations from previous studies [35,36]. From day 103, hormone-producing cell types were detected, including corticotropes, somatotropes, lactotropes, thyrotropes, and gonadotropes. However, TSH, LH, and FSH were present in less than 1% of the total cells. Moreover, this emergence occurred slightly later than reported by Ozone et al., where these cell types were first observed on day 67 and predominantly present by day 84. Additionally, functional assays showed that CRH increased ACTH secretion, while DX suppressed it. Notably, the stem cell markers SOX2+ and SOX2+/CD133+, and a few SOX2+/S100+ cells, were identified, marking the first report of pituitary stem cells in hPSC-derived HP organoids [38].

Next, Taga et al. employed FACS on day 60 to sort EPCAM+ pituitary cells harboring PITX1+/LHX3+ pituitary precursor cells and ACTH-expressing cells. Reaggregated EPCAM+ cells were cultured in gfCDM (Figure 3C) [61] for 30 days to form mature spheres. By day 107, these spheres contained abundant ACTH-expressing cells, along with a few PRL-, GH-, LH-, and FSH-producing cells, and SOX2+/S100+ pituitary stem cells. Furthermore, EPCAM+-sorted spheres exhibited a marked increase in ACTH secretion over long-term culture. Moreover, responsiveness to CRH and DX was maintained. On day 107, EPCAM+ pituitary organoids were collected and transplanted under the renal capsule of hypophysectomized SCID mice. After 24 weeks, the grafts remained intact, containing predominantly ACTH-expressing cells and fewer GH-, PRL-, TSH-, LH-, and FSH-expressing cells. At 4 weeks post-transplantation, basal blood ACTH levels were markedly elevated relative to sham-operated controls. The organoids preserved physiological responsiveness as well, with CRH elevating ACTH secretion and DX decreasing ACTH secretion. Upon administration of lipopolysaccharide, a bacterial endotoxin used to provoke immune activation, ACTH levels rose, substantiating the hormonal responsiveness of the transplanted organoids [73]. Taken together, Taga et al. established the first standalone Ff hPSC-derived pituitary organoid protocol (Table 1) capable of feedback responsiveness pre- and post-transplantation in hypophysectomized mice [38].

Recently, Wang et al. (2025) introduced a revised HP organoid protocol adapted from Ozone et al., with a focus on optimizing *LHX3* expression to improve differentiation efficiency (Figure 3D) [39]. In contrast to Ozone et al., the timing and level of KSR supplementation were altered and the induction medium was supplemented with SB431542 together with LDN193189 to promote pre-patterning toward a neuroectodermal fate. From day 3 onwards, LDN193189 was excluded from the medium to relieve inhibition of the BMP signaling pathway and subsequently facilitate differentiation towards pre-placode ectoderm. Additionally, a novel component, Akt inhibitor VIII, was added to counteract insulin-induced PI3K/AKT signaling, promoting differentiation toward hypothalamic lineages. Furthermore, BMP4, added from day 6 onwards in the protocol, was applied at a higher concentration compared to Ozone et al. Beginning on day 18, BMP4 was excluded from the medium, whereas FGF2 was added to stimulate pituitary identity. On day 30, FGF2 was omitted from the medium, while KSR levels were increased (Figure 3D). The protocol’s efficacy compared to the protocol published by Ozone et al. was evaluated through RT-qPCR and IF staining for the following markers: PITX1, LHX3, ACTH and PRL. Notably, both analyses demonstrated higher expression of all these markers in the optimized HP organoid protocol. The addition of CRH, or changing to a low-glucose medium, led to increased ACTH, whereas DX treatment reduced its secretion. Similar tests were carried out with bromocriptine, resulting in a decrease in PRL secretion. Collectively, these functional assays demonstrate that the corticotropes and lactotropes in the HP organoids can respond to both positive and negative feedback, including intercompartmental communication between CRH+ neurons and corticotropes, as seen in Kasai et al. [39].

Next, Wang et al. used scRNA-seq to uncover the cellular complexity of the HP organoids, and identified a total of nine clusters, including: anterior pituitary endocrine cells (APECs; *PITX1+*, *LHX3+*), neurons (*DCX+*, *STMN2+*, *NEFM+*), non-neural ectoderm (*TFAP2A+*, *TFAP2C+*, *GATA3+*), neural stem and progenitor cells (*SOX9+*, *VIM+*, *SLC1A3+*), ependymocytes (*CCDC153+*), fibroblasts (*TWIST1+*), posterior pituicytes (*OTX2+*), and cycling cells (*MKI67+*) [39]. The neuron cluster comprised a diverse array of neuronal subtypes: glutamatergic, GABAergic, dopaminergic, histaminergic, and cholinergic neurons. In the APECs cluster, corticotrope-specific markers *POMC* and *TBX19* were detected in 33% and 15% of cells, respectively, demonstrating that corticotropes are the major pituitary-hormone-producing cell type present in these organoids. Furthermore, *POU1F1* expression was detected in 11% of APECs, while *PRL*, *TSHβ*, and *GH1* were expressed in only 1.6%, 0.25%, and 0.125% of cells, respectively. Moreover, the gonadotrope and thyrotrope lineages do not yet appear fully matured within the model, evidenced by the expression of the shared α-subunit (CGA) of LH, FSH, and TSH, and only low levels of their respective β-subunits [39].

Additionally, Wang et al. conducted stereo RNA-seq, a method that maps gene expression in tissue with high spatial resolution, revealing four clusters: APECs (*POMC+*, *CGA+*, *PRL+*), neurons (*STMN2+*, *MAP2+*), neural stem and progenitor cells (*VIM+*, *SOX9+*), and low-quality cells. Compared to scRNA-seq, stereo-RNA seq showed higher expression of *POMC*, *CGA*, and *PRL*, whereas lower levels of *TBX19*, *POU1F1*, and *NR5A1* were observed. Importantly, stereo RNA-seq enabled spatial mapping of cell types, revealing a structure that Wang et al. described as resembling a native pituitary gland. Lastly, the potential of the HP organoids as a platform for studying pituitary development was evaluated by examining the role of *SOX3* in this process. Wang et al. downregulated *SOX3* through gene interference, leading to a profoundly decreased expression of *SHH*, *PITX1*, *LHX3*, *POMC*, and *PRL* at later stages of HP organoid development. Interestingly, continuous supplementation of SHH rescued this phenotype partially. Collectively, the data shows that SHH, originating from *SOX3*-expressing neural cells, guides non-neural cells toward pituitary differentiation. Taken together, Wang et al. developed a novel protocol for HP organoid generation that leads to a higher expression of PITX1, LHX3, ACTH and PRL compared to the model generated by Ozone et al., suggesting a higher differentiation efficiency [39]. Nevertheless, low *GH1*, *TSHβ*, *FSHβ*, and *LHβ* expression remains a limitation of the model (Table 1).

A major challenge in generating HP organoids lies in the extended duration of the protocol, resulting in high costs and loss of resources if the organoids fail to adopt HP identity. Therefore, Asano et al. (2024) [74] strived to develop a predictive model that applies deep learning to predict ACTH secretion, used as a marker for successful HP organoid development, based on the organoid area expressing RAX on day 30. Remarkably, the model achieved an accuracy of approximately 70%, outperforming field experts whose accuracy ranged between 46.7% and 60% [74]. One year later, Matsumoto et al. employed a similar approach, developing a machine-learning-based model to predict successful HP organoid differentiation as early as day 9 using phase-contrast imaging. Strikingly, this model achieved an accuracy of 79%, outperforming the previously developed model from 2024 [75].

Current research in pituitary organoid development reflects a dual focus: optimizing organoid models to closely mimic the native pituitary gland and applying these models in disease studies. In 2019, Matsumoto et al. modified the protocol established by Ozone et al. to develop a new model for congenital pituitary hypoplasia, a disease known for impaired pituitary development [76,77,78]. Patient-derived hiPSCs were utilized to investigate the disease, revealing that mutations in the *OTX2* gene, expressed in the hypothalamus, led to increased apoptosis of pituitary progenitor cells and therefore severe impairment of differentiation towards pituitary cell types [76]. Mac et al. (2024) aimed to clarify the role of *NFKB2* in DAVID syndrome, a condition characterized by ACTH deficiency (ACTHD) and immune dysfunction [79,80]. First, Mac et al. introduced a homozygous TBX19^K146R/K146R^ missense pathogenic variant into hiPSCs, an allele associated with the ACTHD [79,81]. Subsequently, the hiPSCs were differentiated into HP organoids using a modified version of the protocol by Matsumoto et al. [76]. The organoids revealed a marked impairment in corticotrope differentiation compared to control organoids, thereby validating the methodological approach. DAVID syndrome is associated with variants in the *NFKB2* gene [79,82,83]. Therefore, organoids were established from hiPSCs-harboring *NFKB2^D865G/D865G^* mutations. The results demonstrated that this mutation disrupts multiple stages of pituitary development, altering gene expression linked to progenitor formation, hypothalamic signaling, epithelial-to-mesenchymal transition, lineage specification, and corticotrope differentiation. Lastly, Kanie et al. (2025) established a human model of anti-PIT1 hypophysitis, an autoimmune disorder affecting hormone secretion from PIT1+ pituitary cells, by generating HP organoids, using a modified version of the protocol by Ozone et al., from patient-derived hiPSCs carrying the HLA haplotype [84,85]. These organoids were cocultured with PIT1-reactive cytotoxic T cells isolated from the patient’s peripheral blood mononuclear cells, enabling direct investigation and providing a novel platform for disease studies and drug development [84].

## 4. Thyroid Gland Organoids

**Figure 4:**
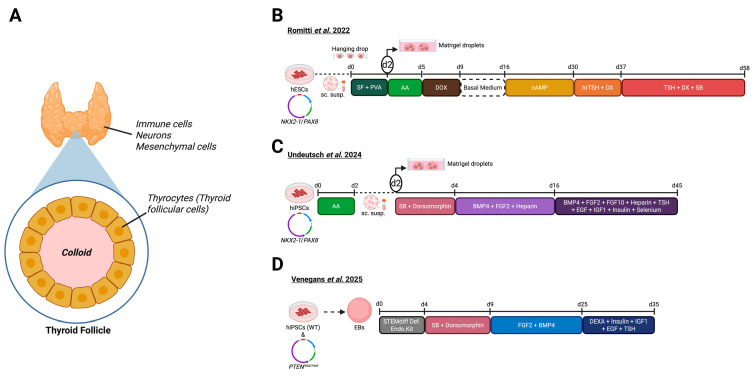
hESC/hiPSC-derived thyroid organoids. (**A**) Anatomy of the human thyroid gland. (**B**–**D**) hESC/hiPSC-based thyroid organoid generation methods developed by Romitti et al. [40] (**B**), Undeutsch et al. [42] (**C**), and Venegans et al. [43] (**D**). (hESC: human embryonic stem cell, hiPSCs: human-induced pluripotent stem cells, d: day, sc. susp.: single-cell suspension, EBs: embryoid bodies, DOX: doxycycline, AA: activin A, DX: dexamethasone, WT: wild-type, STEMdiff Def. Endo Kit: STEMdiff™ Definitive Endoderm Differentiation Kit). Created in BioRender. Vankelecom, H. (2026) https://BioRender.com/tekg613.

The thyroid gland is one of the major glands of the endocrine system, responsible for the regulation of metabolism and growth [5]. This butterfly-shaped gland is located just below the larynx, anterior to the trachea, and between the common carotid arteries. It is composed of multiple cell types, including endocrine cells (primarily thyrocytes), immune cells, neuronal cells, and mesenchymal cells (Figure 4A) [86]. The regulatory function of the thyroid depends mainly on thyrocytes, which form spherical follicles surrounding a colloid-filled lumen, and are therefore also called thyroid follicular cells (TFCs; (Figure 4A)) [87]. The colloid contains thyroglobulin (TG), the essential precursor for thyroid hormone production [88]. Upon TSH stimulation, iodination of TG occurs extracellularly at the apical membrane, where thyroid peroxidase iodinates TG present in the follicular lumen [4]. The resulting iodinated thyroglobulin (TG-I) serves as the storage form of thyroid hormones. When needed, thyrocytes endocytose TG-I from the lumen and, through proteolytic processing, release T4 and T3 into the bloodstream [86]. In peripheral tissues such as the liver, T4 is further converted into the more active T3 [89]. Despite the progress in human thyroid research, much remains unknown about the molecular mechanisms underlying thyroid hormone biosynthesis, human thyroid development, pathophysiology, and the interplay among endocrine axes. So far, models such as primary follicular cells, primary-cell-based thyroid organoids, and rodent models have been essential for thyroid research; however, they either lack human origin or are unable to recapitulate thyroid development [90,91]. 2D differentiation protocols for PSC-derived human thyroid cell types are widely used in thyroid research. Yet, when applied as tools for regenerative therapies, they fail to show substantial therapeutic effects due to incomplete differentiation or the absence of fully matured cell types [92]. Therefore, developing 3D in vitro models that closely recapitulate human thyroid biology and disease is essential. Such models could provide powerful platforms to uncover novel pathways regulating thyroid function and pathology.

The development of 3D human iPSC- and ESC-based thyroid models has only started in recent years. In 2022, Romitti et al. reported for the first time the generation of thyroid organoids from hESCs (HES3 line) (Figure 4B) [40]. Previous protocols had successfully differentiated and matured mouse ESCs into thyroid-like cells; however, when applied to hESCs or hiPSCs, these approaches failed to generate cells with thyroid functionality [93,94,95,96]. Romitti et al. modified a previously established *NKX2-1*^WT/GFP^ knock-in line [97] into an inducible model capable of overexpressing *NKX2-1* and PAX8, key TFs for thyroid lineage specification, upon doxycycline (DOX) treatment. Using this engineered cell line, they generated EBs following earlier protocols [98,99]. After 2 days, approximately, EBs were embedded in Matrigel and the culture medium was supplemented with Activin A (AA) to induce foregut endoderm specification, which was confirmed by elevated *SOX17* and *FOXA2* expression. Subsequent DOX treatment promoted the expression of *NKX2-1* and *PAX8*. Next, from day 9 to 16, cultures were maintained in basal medium to enhance thyroid fate commitment. Bromodeoxyuridine labeling revealed a gradual decline in proliferation, consistent with a reciprocal relationship between proliferation and differentiation during thyroid lineage acquisition, which was further strengthened by cAMP stimulation for the following 14 days. By day 28, early signs of folliculogenesis appeared, characterized by the formation of follicle-like structures with strong expression of *TG* and *PAX8*. Folliculogenesis was further enhanced by the addition of human recombinant TSH and DX, which increased the abundance of key thyroid maturation markers NIS and TPO by day 38. TGF-ß is known to have pro-inflammatory activity that can suppress thyroid differentiation [100]. Therefore, to promote further maturation, the TGF-β pathway was inhibited, using SB431542, from day 37 until the end of the protocol. Although TG+ cells were already visible by day 37, TGF-β inhibition further upregulated *TG*, *TPO*, and *SLC5A5* (i.e., the gene that encodes *NIS*) expression. These cells showed an intra-luminal space and cytoplasmic accumulation of TG and immunostaining revealed *NKX2-1*+/ECAD+ follicular structures. Like TG, TPO was detected both in the cytoplasm and enriched at the apical membrane of *NKX2-1*+ cells. Remarkably, in most follicles, TG-I was detected, confirming the establishment of the biochemical machinery essential for T3 and T4 hormone synthesis [40,101].

scRNA-seq analysis performed on 45-day-old organoids revealed a cluster composed of TFCs, defined by the expression of *NKX2-1*, *PAX8*, *FOXE1*, and *HHEX*. This cluster was further subdivided into three subclusters: thyroid progenitors (only *NKX2-1*, *PAX8*, and *FOXE1*), immature thyrocytes (low levels of *TG* and *TSHR*), and mature thyrocytes (high levels of *TG* and *TSHR*). This transcriptomic analysis of the organoids revealed the presence of non-thyroid cells, including fibroblasts, cardiovascular cells, airway cells, and goblet cells, most probably because *NKX2-1* is an essential gene for forebrain and lung development [97,102]. scRNA-seq analysis performed on 58-day-old organoids showed only immature and mature thyrocytes, suggesting a major role of TGF-ß inhibition on thyroid maturation. On the other hand, the organoids still contain the same non-thyroid cell types [40].

To assess functional capacity, 45-day-old thyroid organoids were transplanted under the kidney capsule of NOD-SCID mice whose thyroid glands were eradicated by radioactive iodine (RAI) treatment, following a low-iodine diet to enhance RAI uptake. Five weeks post-transplantation, researchers observed the formation of organized thyroid follicles and a vascular network surrounding the grafts. Immunostainings revealed *NKX2-1*+ cells exhibiting apical–basal polarization, confirmed by ECAD labeling, while TG accumulated in the follicular lumen and TPO localized primarily to the apical membrane. Nearly 60% of follicles were T4-positive, closely resembling human thyroid morphology and function. T4 and total thyroid hormone plasma levels were increased compared to non-grafted mice, indicating functional rescue at later stages. Furthermore, increased hepatic expression of *Dio1* suggested systemic restoration of thyroid hormone metabolism in organoid-grafted, RAI-treated animals, demonstrating that the organoids achieved physiological functionality in vivo. Overall, Romitti et al. developed a hESC-based thyroid organoid model that contains mature thyrocytes and that can produce thyroid hormones, which rescue hypothyroidic mice (Table 1) [40].

Endocrine-disturbing chemicals (EDCs) are organic or synthetic compounds that can alter or disturb the hormonal balance of fundamental biological processes such as sex hormone biosynthesis [98]. Interestingly, abnormal levels of sex hormones can influence thyroid pathologies such as cancer, hyperthyroidism and autoimmune diseases [103,104,105]. In 2024, Nazarri et al. utilized the Romitti et al. protocol to investigate the effects of EDCs (benzo[a]pyrene (BaP) and PCB153) on human thyrocytes in the context of sex hormone responses [40,41]. After full maturation (>58 days), the organoids were combined into Matrigel droplets (approximately 1200 follicles per droplet) and maintained in serum-free medium for 3 days before initiating hormonal treatments with PG, β-estradiol (E2), and dihydrotestosterone (DHT), in different ratios, to mimic male (20 pg/mL E2, 0.6 ng/mL DHT, 0.5 ng/mL PG) or female (250 pg/mL E2, 0.1 ng/mL DHT, 10 ng/mL PG) hormone conditions, for 4 days. On day 7, EDCs were introduced into the cultures for 24 h, after which the transcriptomic profiles of the organoids were analyzed with scRNA-seq [41]. Transcriptomic analysis revealed the presence of mature thyrocytes, comprising 11% of all cells in organoids exposed to male hormone conditions, and 5.9% of all cells in female hormone-exposed organoids. These mature thyrocyte cells expressed key thyroid markers such as *NKX2-1*, *TG*, and *TPO*, but lacked *SLC5A5* expression. Among thyroid-related genes, only *TG* was significantly affected by male hormonal conditions in BaP-exposed organoids. Interestingly, sex hormone receptor genes (*AR*, *ESR1*, *ESR2*, and *PGR*) exhibited low expression levels across all conditions. Conversely, genes associated with oxidative phosphorylation and lipid metabolism and transport (e.g., *APOA1*, *FABP1*) were enriched in hormone-treated organoids. When male hormonal conditions were combined with BaP, the organoids exhibited a pro-inflammatory transcriptional profile, with upregulation of *NFKB1*, *NFKBIA*, *S100A9*, and *SAA1*, whereas organoids treated with female hormonal conditions showed no comparable enrichment. Overall, Nazzari et al. employed the thyroid organoid model to demonstrate how distinct hormonal contexts modulate thyrocyte responses to EDCs through sc transcriptomic profiling. This study highlights that Romitti’s protocol not only provides a robust platform for functional studies but also serves as a versatile system to investigate inter-endocrine communication (Table 1) [41].

In the same year, Undeutsch et al. (2024) published a completely new thyroid organoid induction protocol using hiPSCs (Figure 4C) [42]. This protocol was primarily based on Kurmann et al., who had previously described a method to generate thyroid follicular organoids from mouse iPSCs [93]. The key innovation of the Undeutsch protocol lies in its use of serum-free conditions, which aims to render the resulting organoids suitable for transplantation into human patients, and in the rapid generation of mature TFCs within 1 month. To trace thyroid lineage specification, the authors engineered a bi-fluorescent reporter iPSC line expressing *NKX2-1*^GFP^ and PAX8^tdTomato(tdT)^, termed BU3 NGP8T. When directed toward the foregut endoderm lineage using AA for 2 to 3 days to enhance efficiency, these cells showed elevated expression of *SOX17*, *FOXA2*, and *T* (*BRACHYURY*) [93,95,106]. The resulting endodermal cells (800–2500 cells/µL) were then embedded in Matrigel and exposed to SB431542 and dorsomorphin for 2 days to achieve dual SMAD inhibition, guiding differentiation toward the anterior foregut endoderm fate. Subsequent exposure to FGF2 and BMP4 further promoted thyroid lineage commitment, consistent with previous reports [93].

This approach yielded approximately 46.7% dual-positive GFP^+^/tdT^+^ cells, showing robust expression of *NKX2-1*, *PAX8*, *HHEX*, and *FOXE1*, indicating efficient generation of thyroid progenitors early during organoid differentiation. Importantly, this population also expressed key TFC markers (*TPO*, *TSHR*, and *TG*), although at lower levels compared to adult human thyroid tissue. From day 12 to 29, organoids were cultured in serum-free differentiation medium supplemented with TSH to promote maturation of TFCs. To assess the role of BMP4 in thyroid differentiation, parallel cultures were maintained with or without BMP4. scRNA-seq analysis at day 29 revealed that BMP4-exposed organoids were more mature, demonstrating that BMP4 signaling is essential for efficient thyroid differentiation and maturation in this system [42].

The thyroid organoids were further characterized both structurally and functionally. On day 45, luminal-like follicular structures, which expressed both *NKX2-1* and PAX8, contained secreted TG in the luminal part. Interestingly, even at day 45, these follicular structures lacked the important thyroid maturation marker SLC5A5 that was present in the organoids obtained with the protocol from Romitti et al. [40]. To understand if this new protocol can be applied to different cell lines, Undeutsch et al. used the C17 iPSC and RUES2 ESC lines. RUES2 line revealed stage- and time-dependent differentiation to thyroid fate with formation of thyroid progenitors and TFCs. On the other hand, C17-derived organoids revealed lower expression of thyroid progenitor markers compared to RUES2 and BU3 NGP8T, suggesting that the protocol needs further optimization when using different cell lines, potentially because of donor variability [107]. The functionality of thyroid organoids was tested by subrenal xenotransplantation of thyroid organoids to hypothyroid mice [40,98]. Twelve weeks after transplantation, renal capsular grafts were visible and the number of double-positive (*NKX2-1* and PAX8) cells in the xenografted organoids increased from 67% to 74% and from 54% to 63% percent, for 30- and 47-day-old organoids, respectively. The grafts revealed the expression of human-specific nuclear mitotic apparatus and TG, but not of T4. Even though follicular structures revealed matching morphology with human tissue and vascularization, they did not result in increased plasma levels of T4 in hypothyroid mice. These findings, combined with the lack of *SLC5A5* expression in the organoids, suggest that the organoids are not mature enough to secrete the thyroid hormones necessary to rescue hypothyroidism. The authors suggest that inhibition of the MAPK/PI3K pathway together with stimulating the TSH/cAMP pathway might increase the expression of *SLC5A5*. To have proof of concept, they inhibited MEK1, a key regulator of the MAPK/PI3K pathway, in maturing TFCs with PD98059, and induced cAMP by adding cAMP and IBMX from day 26 onwards, which resulted in time-dependent upregulation of *SLC5A5* expression [42].

To summarize, in this study a new serum-free protocol for transplantable human organoids was proposed (Table 1). They revealed the major contribution of BMP4 to thyroid differentiation. On the other hand, the organoids are still immature, as exhibited by the lack of T4 and T3 secretion, which are crucial for studying the interaction between the thyroid and other organs, as well as to be used as a potential treatment strategy in patients suffering from hypothyroidism [42].

In 2025, Venegas et al. developed a protocol to generate both healthy thyroid organoids and tumoroids to study PTEN hamartoma tumor syndrome, an autosomal dominant genetic disorder caused by mutations in the *PTEN* tumor suppressor gene, leading to increased risk of benign (hamartomas) and malignant tumors (Figure 4D) [43]. For this study, researchers generated five PTEN-mutant lines containing two different mutant PTEN alleles M134R and G132D. The organoid induction process began with EB formation from iPSCs, cultured in StemFlex medium for 24 h, followed by treatment with a commercial endoderm induction kit until day 4. The resulting EBs were then cultured in anterior endoderm medium, supplemented with dorsomorphin and SB431542 (TGF-β inhibitor) for 5 days, leading to the formation of anterior endoderm cells, expressing AFP and PAX9. Subsequently, organoids were exposed to FGF2 and BMP4 from day 9 to 25, promoting differentiation into *NKX2-1*+ thyroid precursor cells. Moreover, immunostainings performed on day 25 showed TTF1 expression, an essential TF for the formation of primitive thyroid, indicating thyroid fate specification of the obtained organoids. To drive maturation, cultures were supplemented with DX, insulin, IGF-1, EGF, and TSH until day 35. This treatment regimen resulted in organoids exhibiting strong expression of TPO, TSHR, and TG by day 35 (Table 1). ELISA assays performed on organoid culture media demonstrated that these organoids produced T4 in wild-type (WT/WT) cells. Interestingly, organoids induced from iPSCs carrying the M134R allele showed a similar T4 production, whereas organoids with the G132D mutation secreted more T4. The researchers applied the model to study the effects of irradiation on p53 activation and PTEN function. Radiation treatment of tumoroids revealed that the p53 pathway was strongly upregulated in G132D organoids, and not in M134R organoids. This demonstrates that thyroid organoids can also be used to investigate DNA damage responses and to uncover molecular pathways underlying thyroid tumorigenesis [43].

## 5. Adrenal Gland Organoids

**Figure 5:**
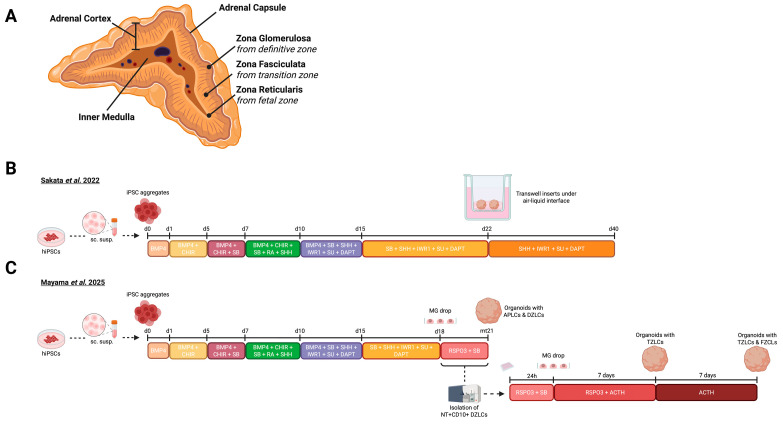
hiPSC-derived adrenal gland organoids. (**A**) Anatomy of the human adrenal gland. (**B**,**C**) hiPSC-based adrenal gland organoid generation methods developed by Sakata et al. [44] (**B**) and Mayama et al. [45] (**C**). (hiPSCs: human-induced pluripotent stem cells, d: day, sc. susp.: single-cell suspension, APLCs: adrenal-primordium-like cells, MG drop: Matrigel droplets, DZLCs: definitive-zone-like cells, TZLCs: transition-zone-like cells, FZLCs: fetal-zone-like cells, RA: retinoic acid, SHH: sonic hedgehog). Created in BioRender. Vankelecom, H. (2026) https://BioRender.com/nlieogo.

Adrenal glands are located just above and medial to the kidneys, and they are crucial for regulation of blood pressure, stress response, and the maturation of gonads. Adrenal glands secrete steroid hormones and catecholamines that are essential for the body’s rapid response to stress [7]. Comparable to the pituitary, adrenal glands also consist of two distinct parts: the adrenal cortex (endocrine-like) and the inner medulla (sympathetic-ganglion-like) (Figure 5A) [108]. The adrenal cortex consists of three main zones that are specialized in distinct types of steroidogeneses. Basically, in the adrenal cortex, cholesterol is catalyzed by cell- or zone-dependent steroid hydroxylase enzymes through serial conversions known as steroidogenesis [109]. Adrenal glands can produce a variety of steroid hormones thanks to their distinct tissue organization: the *zona glomerulosa* (zG) develops from the definitive zone, the *zona fasciculata* (zF) originates from the transition zone, and *zona reticularis* (zR) is derived from the fetal zone (Figure 5A) [110]. zG cells are located just below the adrenal capsule and are essential for aldosterone production [111]. Beneath the zG, large lipid-rich zF cells form a thick layer and are responsible for the secretion of glucocorticoids (cortisol and cortisone) [112]. Below the zF layer, zR cells are located; these cells are essential for the secretion of androgens such as dehydroepiandrosterone (DHEA), DHEA-sulfate (DHEA-S) and androstenedione [113]. These adrenal hormones serve as essential substrates for sex hormones, including estrogens and testosterone, and can also influence metabolism and regulate social behavior [113,114]. At the very core of the gland lies the inner medulla, which consists predominantly of chromaffin cells that produce the neurotransmitters norepinephrine and epinephrine [108].

In recent years substantial advances in thyroid biology have been made; however, much remains to be explored for the human adrenal cortex. Therefore, hESC- and hiPSC-based adrenal cortex organoids are crucial for addressing this gap. In 2022, Sakata and Sasaki et al. developed fetal-zone adrenal-cortex-like organoids for the first time using hiPSCs (Figure 5B) [44]. To track developmental stages, they generated a WT1-p2A-EGFP (WG) line in which GFP is expressed when cells transition to the posterior intermediate mesoderm (PIM) lineage and start to express WT1, as well as a NR5A1-p2A-tdTomato (NT) line, which becomes active when cells enter the adrenocortical progenitor fate, which is marked by NR5A1. They used a previously established 3D protocol for kidney organoids, since both adrenal gland and kidney originate from PIM, with adaptations to optimize the induction towards adrenal gland fate [115]. On day 0, researchers created hiPSC aggregates, which were grown in medium supplemented with BMP4 until d15, to enhance adrenal differentiation, marked by *OSR2*. From day 1 to 10, aggregates were treated with CHIR to stimulate the WNT pathway, supporting the formation of WT1+ PIM-like cells. Starting on day 10, WNT and FGF9 were inhibited by IWR1 and SU-5402, respectively, resulting in enhanced numbers of NR5A1+ cells. To further upregulate *NR5A1* expression, NODAL and activin pathways were inhibited by SB431542 from day 7 to 22. SHH is essential for the formation of human adrenal cortex, whereas DLK1 (a membrane protein that inhibits NOTCH signaling) is highly expressed in the fetal adrenal cortex. To recapitulate these cues, Sakata et al. added SHH (from day 7) and DAPT (a NOTCH inhibitor, from d10) to the medium. This approach decreased nephrogenic cell numbers, and kidney (*PAX2* & *SIX2*) and gonad markers (*LHX9* & *GATA4*) were undetectable at day 22. This time-dependent induction strategy led to scattered expression of CYP11A1, CYP17A1, and SULT2A1, indicating gradual steroidogenic activity. On day 22, organoids were transferred from floating cultures to an air–liquid interface (ALI) system, with the expectation that improved nutrient access and oxygen diffusion to the organoid core would enhance the formation of fetal-zone-like cells (FZLCs) and upregulate definitive zone-like cell (DZLC) markers, such as *NOV* and *HOPX*. This strategy increased the expression of steroidogenesis genes while maintaining NR5A1 protein levels. Histological examination of 28-day-old organoids showed compact cell clusters with spherical nuclei, cytoplasmic vacuoles, and granular morphology, highly resembling the prenatal human fetal zone (FZ), further corroborated by high expression levels of SULT2A, FDX1, FDXR, CYP11A1, and CYP17A1. Interestingly, the organoids could produce IGF-2, which is an essential factor for adrenal cortex development and a promising mediator of inter-organ communication [116]. Bulk RNA-seq and scRNA-seq were performed at different time points and compared to in vivo human adrenal gland datasets [117], showing strong similarities, including the presence of *RSPO3*- and *PDGFRA*-positive capsule-like cells in the organoids, which resembled human adrenal capsule cells closely (Table 1) [44].

The presence of steroidogenic enzymes is crucial for studying adrenal functionality. Sanaka and colleagues measured substantial levels of DHEA and DHEA-S (Δ5 adrenal steroids) in their organoid supernatant; Δ4 steroids (PG, 17-OH PG, cortisol, and aldosterone) on the other hand remained low and androstenedione showed intermediate levels. Interestingly, DHEA peaked on day 27, while DHEA-S levels gradually increased until day 42. Treatment of adrenal gland organoids with ACTH resulted in increased rates of pregnenolone (PG precursor), DHEA, and DHEA-S, showing that the organoids can respond to pituitary-derived signals, making them a promising model for future studies of the hypothalamus–pituitary–adrenal gland axis. Finally, to test whether Δ5 steroidogenesis can be pharmacologically inhibited, the organoids were separately treated with aminoglutethimide (CYP11A1 inhibitor), abiraterone (suppressor of CYP17A1), and orteronel (to inhibit 17,20-lyase), which resulted in inhibition of pregnenolone, DHEA, and DHEA-S production. These results show that the model has the potential to be used as a drug screening platform for steroidogenesis [44].

In mice, NR5A1 plays a critical role in adrenal fate and the regulation of steroidogenesis [118]. To investigate whether NR5A1 has a similar role in human fetal adrenal cortex development, researchers generated an NR5A1 knock-out hiPSC line. The organoids derived from this line showed increased apoptosis rates and downregulation of steroidogenesis. Bulk RNA-seq analysis revealed that the expression of genes involved in early adrenocortical development was not affected, suggesting that early-human fetal cortex development is driven by NR5A1-independent genes [44].

Despite this breakthrough, the Sasaki group aimed to further improve their model and investigate the biological mechanisms underlying human adrenal cortex development (available on bioRxiv; Mayama et al. 2025) (Figure 5C) [45]. To further increase the in vivo relevance of adrenal cortex organoids, Mayama and Sasaki began maintaining the organoids in Matrigel droplets rather than in ALI culture (from day 21), which increased the proportion of FZLCs from 36.8% to 58.6%. Furthermore, they identified that DZLCs (CD10+/NR5A1+) exhibited upregulated expression of WNT pathway members (*LEF1*, *AXIN2*). Moreover, they demonstrated that RSPO3+/NR5A1 capsule-like cells are the source of WNT signaling (*RSPO3*, *GLI1*, *GLI2*), suggesting a positive effect of these cells in promoting the differentiation of DZLCs from adrenal-primordium-like cells. This differentiation can be further enhanced by inhibition of ACTIVIN, NODAL, and NOTCH pathways. The strong resemblance of DZLCs to the definitive zone (DZ) was confirmed by immunostainings, which revealed the presence of essential markers STAR, CYP11A1, CYP17A1 and SULT2A1 (Table 1). PCA and unsupervised hierarchical clustering analysis of scRNA-seq data revealed close clustering of DZLCs with in vivo DZ cells, providing additional evidence for the improved fidelity of adrenal gland organoids [45].

Previously, it was hypothesized that human DZ cells may contribute to the homeostasis of the prenatal adrenal cortex through trans-differentiation into steroidogenic cells and through self-renewal, a process that in mice is driven by ACTH [119]. To investigate whether this mechanism is conserved in the human adrenal gland, adrenal cortex organoids were treated with TZ medium (containing ACTH), which resulted in DZLCs differentiating into transitional-zone-like cells (TZLCs), accompanied by increased expression of HSD3B2 and CYP17A1 (Table 1). Most interestingly, this treatment elevated the cortisol production in the organoids seven-fold. These findings suggest that adrenal cortex organoids do not only sense ACTH signals but also respond to them by modulating cortisol levels [45]. This demonstrates that the adrenal cortex organoid model is valuable for studying both glucocorticoid synthesis and communication within the HP–adrenal cortex axis [45].

To test in vivo functionality of the adrenal gland organoids, Mayama and colleagues produced aggregates from DZLCs and adrenal-primordium-like cells, isolated from the organoids, which were then grafted in the kidney capsule of immunodeficient (NCG) mice. Grafts were collected 50 days after transplantation and analyzed using scRNA-seq. Both types of grafts showed a high resemblance to prenatal and adult human adrenal cortex. Immunostaining revealed the expression of adrenal markers (CD10 for DZLCs, HSD3B2 for TZLCs, and SULT2A1 for FZLCs). However, zonation between the regions was not clear. Strikingly, ACTH treatment of the grafted mice resulted in a significant increase in DHEA-S and cortisol serum concentrations, indicating that the grafts remained responsive to ACTH in vivo [45].

## 6. Ovary Organoids

**Figure 6:**
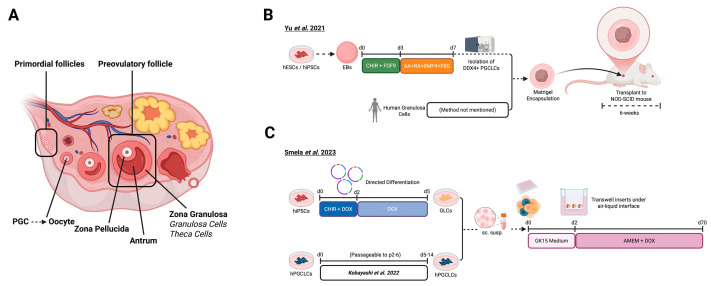
hESC/hiPSC-derived ovary organoids. (**A**) Anatomy of the human ovary. (PGC: primordial germ cell) (**B**,**C**) hESC/hiPSC-based ovary organoid generation methods developed by Yu et al. [46] (**B**) and Smela et al. [47] (**C**). (hESCs: human embryonic stem cells, hiPSCs: human-induced pluripotent stem cells, d: day, sc. susp.: single-cell suspension, EBs: embryoid bodies, NOD-SCID: immune deficient mice, DOX: doxycycline, AA: activin A, RA: retinoic acid, GLCs: granulosa-like cells, p: passage, hPGCLCs: primordium-germ-cell-like cells, AMEM: alpha minimum essential medium). Created in BioRender. Vankelecom, H. (2026) https://BioRender.com/iiyad8k.

The HP–gonad axis is essential for sexual maturation, reproduction and the regulation of body homeostasis [120]. In women, HP–gonadal axis communication circulates through the ovaries, which have two major functions: folliculogenesis followed by oocyte maturation and acting as an endocrine center, producing E2 and PG (Figure 6A) [121]. Folliculogenesis is a systematic, time-dependent process involving the development of primordial follicles and their maturation into preovulatory follicles [122]. Even at neonatal stages, human ovaries already contain numerous primordial follicles, each composed of an immature oocyte surrounded by a single layer of granulosa cells (GCs) that support and protect it [123]. During puberty, FSH and LH signals, released from pituitary gonadotropes, promote the maturation of primordial follicles into more advanced stages, in which a mature oocyte is surrounded by a glycoprotein-rich liquid, known as the *antrum*, and a glycoprotein-based membrane called the *zona pellucida* [124]. The outer region of the *zona pellucida* is enveloped by multiple layers of GCs, which are in turn surrounded by theca cells (forming the *zona granulosa*; Figure 6A) [125]. Throughout oocyte maturation, LH signaling stimulates androstenedione production in the theca cells via CYP17A1-mediated steroidogenesis [126]. Concurrently, under the FSH effect, androstenedione secreted by theca cells is converted into E2 by GCs through the action of the aromatase enzyme CYP19A1 [127]. E2 not only induces endometrial growth but also influences multiple physiological processes, including metabolism, maintenance of muscle mass and behavior [128]. Following follicular rupture during ovulation, GCs further differentiate into granulosa lutein cells, forming *corpus luteum* [129]. These granulosa lutein cells secrete high levels of PG, which prepares the uterus for potential pregnancy [124]. 

Studies on mammalian ovary development date back to the 17th century with the work of Regnier de Graaf and continue to this day, revealing essential developmental mechanisms. One of the major milestones in the study of ovarian development was the identification of primordial germ cells (PGCs), which are the precursors of both sperm and oocytes [130]. Human PGCs appear as early as the fifth week of pregnancy and are characterized by the expression of markers such as DAZL, DDX4, and TFAP2C [131,132,133]. Toward birth, the PGC population expands dramatically, increasing from approximately 500 cells to 6–7 million cells, a pool that is essential for the formation of primordial follicles [133]. To date, numerous protocols have been published for differentiating PGCs from human PSCs; however, none have succeeded in generating a fully human ovarian organoid model that faithfully recapitulates ovarian morphology and folliculogenesis [134,135,136,137,138,139]. This limitation likely reflects the unneglectable role of ovarian somatic cell types in folliculogenesis. Among these somatic cells, GCs emerge around the seventh week of pregnancy and, through paracrine- and juxtacrine-signaling interactions, provide essential support for follicle formation and oocyte maturation [140]. Therefore, the integration of both GCs and somatic cell populations represents a critical requirement for the development of physiologically relevant ovarian models, a concept that has only recently been embraced by the field.

Yu et al. proposed a complex aggregate-based model composed solely of human cells (Figure 6B) [46]. Although this system does not yet represent a self-organizing ovarian organoid, the authors successfully achieved the formation of primordial-follicle-like structures following transplantation into NOD-SCID mice. Their protocol began with the generation of EBs from hESCs (H9 and NTU1, both female lines) or from an umbilical cord blood-derived iPSCs line, which was generated by the authors. EBs were maintained with bFGF in feeder- and serum-free, non-adherent culture conditions for 3 days. Based on evidence from mouse studies and 2D human culture systems, which demonstrates an essential role for AA and retinoic acid (RA) in PGC differentiation [141], the authors incorporated AA and RA into their differentiation strategy. From day 3 to 7, EBs were treated with AA, BMP4, RA, and 15% FBS, resulting in the generation of primordial-germ-cell-like cells (PGCLCs), expressing high levels of *DDX4*, *SSEA1*, *OCT4*, and *SOX17*, although *BLIMP1* (a regulator of PGC identity) expression was absent. Notably, PGCLCs derived from hESCs exhibited higher expression of *SYCP1*, *SYCP2*, and *SYCP3*, all three key regulators of meiotic initiation, compared with iPSC-derived PGCLCs. Immunostaining revealed that SSEA1 expression did not colocalize with DDX4, suggesting distinct temporal expression patterns during PGCLC maturation. Furthermore, compared to hESCs and hiPSCSs, isolated DDX4+ cells showed enrichment of gene ontology terms related to organ and tissue development, and cell migration, together with Western blot results, showed enrichment of oocyte- and folliculogenesis-associated markers, such as DDX4 and ZP3, indicating that these cells may represent a more mature ovarian fate. To further assess the developmental potential of DDX4+ PGCLCs, researchers combined them with human GCs to provide a supportive microenvironment for oogenesis [142]. PGCLCs and GCs were transplanted into NOD-SCID mice, either separately or as combined as aggregates, to evaluate their folliculogenic capacity. Remarkably, after 6 weeks, only the PGCLC-GC aggregates formed follicle-like structures containing a germinal-vesicle-like inner region, expressing DDX4 (possibly an immature oocyte), surrounded by follicle-like layers composed of AMHR2+ cells, representing the transplanted GCs. Taken together, Yu et al. presented a protocol that advances human PGCLCs toward folliculogenesis by demonstrating their capacity to form follicle-like structures and the essential role of GCs in vivo (Table 1) [46]. However, this system remains far from a fully self-organizing ovarian organoid model, as it still requires exogenous human GCs and an in vivo carrier to promote folliculogenesis. Moreover, the model lacks complex and mature ovarian structures, such as the *zona granulosa*.

The term ‘ovaroid’ was first described by Smela et al. in 2023 (Figure 6C) [47]. Although this platform does not yet represent a self-forming ovarian organoid, all cell types used in the study were derived exclusively from either hESCs or hiPSCs. Their strategy involved combining hPGCLCs, generated by Kobayashi et al. [139], from the ATCC-BXS0115 iPSC line, with granulosa-like cells (GLCs), produced through directed differentiation from multiple hiPSC lines. Notably, this directed differentiation of GLCs represents a significant advance, as no previous study had successfully differentiated this cell type in vitro. To achieve this, the authors first predicted candidate TFs essential for GC identity by integrating information from published human and mouse transcriptomic datasets [134,143,144]. This integrative approach resulted in a list of 35 candidate TFs. For controlled TF expression, the researchers employed a DOX-inducible promoter system and introduced TF constructs into multiple iPSC lines, carrying the T2A-tdTomato reporter in the *FOXL2* locus, a gene whose expression is restricted to GCs [145]. Activation of WNT signaling using CHIR during the first 2 days of differentiation, combined with DOX treatment for 5 days, led to the emergence of a small population of *FOXL2+* (tdTomato+) cells. This FOXL2+ population was isolated by FACS, and pooled barcode screening identified *NR5A1*, *RUNX1*/*RUNX2*, *TCF21*, and *GATA4* as the top five enriched TFs. To determine which TF combinations were sufficient to induce GLC identity, different combinations of these factors were introduced into hiPSCs. The authors concluded that *NR5A1* combined with *RUNX1* and/or *RUNX2* was sufficient to generate GLCs, based on FOXL2 and EPCAM expression levels, E2 production capacity, and responsiveness to FSH. Further transcriptomic characterization revealed enrichment of gonadal and granulosa cell markers, including *AMHR2*, *FSHR*, and *IGFBP7*, as well as the ovarian stromal cell marker, *NR2F2*. However, the expression of *WT1*, a regulator of ovarian follicle development, was higher than that observed in vivo [146]. Functionally, these directly differentiated GLCs can respond to FSH and exhibit steroidogenic activity (conversion of androstenedione into E2), although the strength of this response varied depending on the TF combination used. Notably, the *NR5A1*/*TCF21* combination yielded the highest E2 production (~7 ng/mL) in the presence of FSH and androstenedione, whereas NR5A1/RUNX2 resulted in the lowest levels (~1 ng/mL). Interestingly, GLCs were also capable of secreting PG at substantially higher levels in the absence of androstenedione [47].

After thoroughly characterizing the GLCs, Smela et al. generated the first iPSC-based human ovaroids. To achieve this, they combined 100.000 GLCs with 10.000 PGCLCs per well in a low-binding U-bottom plate. The authors argued that this assembly strategy was critical for the maturation of PGCLCs, because it promotes the expression of DAZL, a factor essential for gametogenesis [147]. Ovaroids were maintained in GK15 medium for 2 days, after which five to six ovaroids were transferred onto collagen-coated Transwell inserts and cultured under ALI conditions and supplemented with KSR, DOX, and 2-mercaptoethanol. Expression of the maturation marker DAZL was already detectable by day 4 in a subpopulation of OCT4+ PGCLCs, with maximal numbers of DAZL+ cells observed at day 14. Notably, by day 16, a DAZL+/OCT4− population emerged, mimicking human in vivo oogonia during ovarian development [148], accompanied by decreased expression of the early PGC marker TFAP2C. This degree of PGCLC maturation was not observed in the earlier aggregate model proposed by Yu et al. However, DAZL localization remained restricted to the nucleus whereas it should have been visible in cytoplasm, suggesting that post-translational modifications required for full DAZL activity may not have occurred (yet). Remarkably, the authors observed the formation of primordial-follicle-like structures composed of AMHR2+/FOXL2+ GLCs at the same stage, even in the absence of oocytes. Folliculogenesis progressed up to day 70, resulting in follicles of varying sizes. Importantly, some structures exhibited a thick multilayer of GLCs resembling a *zona*-*granulosa*-like architecture with a central *antrum*, recapitulating the later stages of folliculogenesis, a feature not observed in the aggregates developed by Yu et al. Moreover, NR2F2 immunostaining indicated that these follicle-like structures were surrounded by stromal cells. To assess steroidogenic functionality, ovaroids were exposed to androstenedione and FSH. ELISA analyses revealed robust production of E2 (average 28.6 ng/mL) and PG (average 24.5 ng/mL), depending on the TF combination used to induce GLCs. E2 production was dependent on androstenedione and further enhanced by FSH (an approximately 30% increase), whereas PG secretion occurred across all conditions and was highest in the absence of androstenedione [47].

scRNA-seq analysis of ovaroids at days 2, 4, 8, and 14 revealed that the predominant cell population consisted of GLCs expressing *CD82*, *WNT4*, and *FOXL2*. Within this cluster, a subpopulation corresponding to antral GCs was identified based on expression of *CYP19A1* and *FSHR*. One of the smallest clusters comprised *NR2F2*-expressing stromal cells that lacked *CYP17A1*, indicating the absence of theca cells, an essential component for proper ovarian follicle development [149]. A *PGCLC*+ cluster expressing *CD38*, *KIT*, and *PRDM1* was also detected. Notably, this cluster showed enrichment of *XIST* and *TSIX* expression, suggesting initiation of X-chromosome reactivation. Mapping of the ovaroid dataset onto a human fetal ovarian reference atlas [148] revealed a maturating cellular composition, while lacking the non-ovarian cell types observed in vivo. Taken together, Smela et al. generated the first human ovaroid model, derived solely from iPSCs, which exhibits functional steroidogenesis, responds to FSH stimulation by secreting E2 and PG, forms prominent *zona*-*granulosa*-like structures, and displays a transcriptomic profile closely resembling that of the human fetal ovary (Table 1) [47]. Nevertheless, this model does not yet represent a fully self-assembling organoid, as it relies on the aggregation of PGCLCs and GLCs, depends on directed differentiation of GLCs, and lacks essential ovarian cell types such as theca cells.

## 7. Testis Organoids

**Figure 7:**
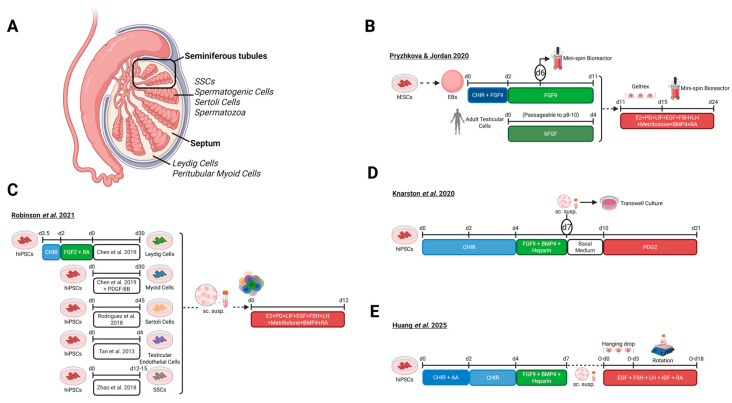
hESC/hiPSC-derived testis organoids. (**A**) Anatomy of the human testis. (SSCs: spermatogonial stem cells) (**B**–**E**) hESC/hiPSC-based ovary organoid generation methods developed by Pryzhkova and Jordan [48] (**B**), Robinson et al. [49] (**C**), Knarston et al. [50] (**D**), and Huang et al. [51] (**E**). (hESCs: human embryonic stem cells, hiPSCs: human-induced pluripotent stem cells, d: day, O-d: organoid day, sc. susp.: single-cell suspension, EBs: embryoid bodies, DOX: doxycycline, RA: retinoic acid, AA: activin A, FSH: follicle-stimulating hormone, LH: luteinizing hormone, E2: estradiol, PG: progesterone). Created in BioRender. Vankelecom, H. (2026) https://BioRender.com/my7896g.

In females, hypothalamic–pituitary–gonadal communication is mediated through the ovaries, whereas in males the testes form the central effector organ of this neuroendocrine axis. Beyond generating male gametes, the testes produce androgens, which are essential for systemic physiological homeostasis [12]. Spermatogenesis, the process of male gamete production, is a highly coordinated developmental program, beginning with spermatogonial stem cells (SSCs) and progressing through successive stages of germ cell maturation to form spermatozoa [150]. SSCs and their differentiating progeny reside within the seminiferous epithelium of the seminiferous tubules, the primary site of sperm production [151]. These tubules contain both spermatogenic cells and Sertoli cells, which provide structural, metabolic, and paracrine support essential for germ cell development (Figure 7A). Sertoli cell activity is regulated by pituitary-derived FSH and, in turn, Sertoli cells secrete inhibin to negatively regulate FSH release (Figure 1D) [151]. Sertoli cells also play a critical role during embryogenesis, producing anti-Müllerian hormone (AMH) to drive male sexual differentiation [152]. Seminiferous tubules are encased by layers of peritubular myoid cells, which exhibit smooth-muscle-like contractile properties and contribute to the extracellular matrix production that supports the seminiferous epithelium (Figure 7A) [153]. In the interstitial compartment, Leydig cells synthesize testosterone from DHEA, via the enzyme HSD3B, under pituitary-derived LH regulation (Figure 1D) [154]. Testosterone is indispensable for normal spermatogenesis and promotes secondary sexual characteristics and bone and muscle development [12].

Studying human testicular biology is essential for understanding the underlying mechanisms governing testis function, which requires robust human research models that closely mimic the in vivo testicular environment. In 2020, Pryzhkova and Jordan reported the first hESC-based testis-like spheroids (Figure 7B) [48]. Like the ovaroid models developed by Yu et al. and Smela et al., their approach involved combining hESCs with adult testicular cells to promote further maturation and to generate a more physiologically relevant 3D environment. The researchers first optimized adult testicular cell culture conditions by replacing FBS with bFGF to match serum-free conditions used for hESCs. Adult testicular cells cultured with bFGF displayed growth rates comparable to FBS-cultured cells, showed no detectable differences in SOX9, WT1, or STAR expression, and maintained robust expression of Sertoli cell (SOX9, WT1, AMH) and Leydig cell (IGFBP3, VIM, STAR) markers even after eight passages. To generate testis-like spheroids, EBs were generated and directed toward a pre-Sertoli fate using the WNT activator CHIR during the first 2 days of differentiation. In parallel, EBs were continuously treated with FGF9 from day 0 to enhance SOX9 expression, promote male gonadal specification, and prevent intermediate mesoderm progenitors from adopting a kidney fate [155]. On day 6, EBs were transferred to a mini-spin bioreactor and maintained until day 28. By day 10, spheroids exhibited tubular-like structures, which were confirmed by histological examination. IF analysis demonstrated that these tubular-like structures were lined by SOX9+ cells, although SOX9+ were also distributed throughout other regions of the spheroids. Given that endothelial cell migration from the adjacent mesonephros is essential for testis cord development [156], the authors examined PECAM1 expression and observed colocalization with SOX9+ cells. Moreover, WT1 IF staining showed the presence of gonadal progenitors located outside of the tubular-like structures. To further enhance testicular maturation, day 11 spheroids were combined with adult testicular cells in a Geltrex extracellular matrix and cultured for 4 days to generate aggregates. These aggregates were subsequently transferred back to the mini-spin bioreactor and maintained until day 24 (Figure 7B). Overall, Pryzhkova and Jordan established a testis-like spheroid model containing pre-Sertoli cells (SOX9+) and gonadal progenitors (WT1+) capable of forming tubular-like structures and integrating with adult testicular cells (Table 1) [48]. However, this model lacks comprehensive characterization of additional testicular cell types, does not report on the maturation status of the generated structures, provides no evidence of spermatogenesis, and omits functional assays demonstrating hormone production.

Robinson et al. adopted a different strategy by inducing multiple testicular cell types from hiPSCs and assembling them into aggregates to recapitulate human testicular architecture (Figure 7C) (available on bioRxiv) [49]. To induce Leydig cells, the researchers used a previously published differentiation protocol with minor optimizations [157]. Specifically, hiPSCs were treated with CHIR for 36 h, followed by 2-day treatment with FGF2 and RA to induce an intermediate mesenchymal fate. This modification reduced mis-differentiation into undesired lineages and increased the yield of Leydig cells. The differentiated Leydig cells expressed the testicular marker GATA4 and the differentiation marker NESTIN, indicating an immature phenotype. Nevertheless, the expression of HSD3B suggested potential steroidogenic activity. To generate peritubular myoid cells, the Leydig cell differentiation protocol was further adapted by replacing PDGF-AA with PDGF-BB, resulting in enrichment of the myoid cell marker MYH11. Sertoli cells were generated using the protocol described by Rodriguez et al. [158], with feeder layers replaced by Matrigel-coated plates. These Sertoli-like cells showed positive IF staining for SOX9 and GATA4, while the mature Sertoli cell marker, GATA1, is only present in a small population. Differentiation of testicular endothelial cells and SSCs was done according to a published protocol without further optimization [49,159,160].

After differentiation of individual testicular cell types, cells were aggregated using the AggreWell800 system and, 1 day later, transferred to suspension culture for an additional 12 days. During this period, the aggregates were maintained in a growth-factor- and hormone-rich medium containing E2, PG, LIF, EGF, FSH, LH, the anabolic steroid metribolone, BMP4, and RA, all of which are necessary to mimic the in vivo human testicular environment [161,162]. H&E staining revealed that these aggregates lacked tubular-like structures, which had been reported before by Pryzhkova and Jordan [48]. Notably, expression of *DAZL*, an essential factor for both SSCs and PGCs [132], was elevated in aggregates, together with the meiotic marker *SYCP3* and spermatogenesis-associated markers *TNP1* and *PRM2*. The presence of PRM2+ cells, a marker of the post-meiotic stages, suggests a potential initiation of spermatogenesis within these organoids. Moreover, IF staining revealed that subsets of cells were positive for either INSL3 or GATA1, which are maturation markers for Leydig and Sertoli cells, respectively. In conclusion, Robinson et al. proposed an alternative strategy in which distinct testicular cell types are differentiated separately and subsequently assembled into aggregates, resulting in structures that display features suggestive of early spermatogenesis and a complex cellular organization more closely resembling the human testis (Table 1). The lack of tubular structures and, most importantly, the absence of functional assays assessing sperm production or endocrine activity, limits the conclusions that can be drawn regarding full testicular functionality [49].

The first complex, nearly self-organizing testicular organoids derived from hiPSCs that were well characterized and showed signs of steroidogenic capacity were reported by Knarston et al. in 2020 (Figure 7D) [50]. The protocol begins with treatment of male iPSC lines with CHIR for 4 days to induce posterior primitive streak identity and reduce expression of the pluripotency marker *OCT4*. The cells were then directed toward intermediate (marked by *FOXF1)* and lateral plate mesoderm (marked by *LHX1* and *PAX2*) by culturing them with FGF9, heparin, and BMP4 until day 7. From day 7 to day 10, cells were maintained in basal medium, leading to the emergence of bipotential gonadal cells expressing *GATA4*, *WT1*, *LHX9*, and *EMX2*, as well as Sertoli-like cells expressing *SOX9*, *FGF9*, and *AMH*. The authors proposed that day 7 represents the optimal time point for testicular organoid generation, based on mouse studies demonstrating that testis-like structures (derived from reaggregating isolated urogenital ridges) can only be formed during a limited developmental window [163,164]. Cells harvested at this stage were cultured on Transwell inserts, following the protocol of Takasato et al. [165], and maintained with only basal medium for 3 days. Starting from day 10, the cultures were supplemented with prostaglandin D2 (PDG2), a factor known to enhance *SOX9* expression. Consistent with previous observations, PDG2 supplementation also increased the expression of *AMH* and *HSD3B*. The organoids were maintained until day 21, at which point they reached nearly 3 mm in diameter and displayed prominent tubular-like structures [50].

By day 21 of organoid culture, expression of Sertoli cell markers and NR5A1 was increased, a pattern not observed in matched monolayer cultures. Markers of bipotential gonadal cells (WT1, GATA3, LHX9) and Leydig cells (STAR, CYP17A1, HSD3B) were also increased. IF analysis revealed that the Sertoli marker SOX9 was strongly concentrated within tubular-like regions. In contrast, the bipotential marker GATA3 was broadly distributed throughout the organoids, with higher intensity along the tubular structures. Cells co-expressing SOX9 and GATA4 were primarily located in the inter-tubular spaces, and WT1 displayed a distribution similar to GATA4, including a small subset of WT1^+^/AMH^+^ cells. Cytosolic HSD3B was detected within cells in the tubular-like regions, indicating the onset of testicular steroidogenesis, and a subset of cells expressed the Leydig cell marker STAR. Altogether, Knarston et al. established a highly organized and well-characterized testicular organoid model, that, although requiring an initial monolayer induction step, shows partial self-organization and demonstrates potential for steroidogenic activity (Table 1) [50].

The human testicular organoid models discussed so far have lacked two major experimental components: (1) functional assays assessing responsiveness to hormonal signals or steroidogenic capacity, and (2) in-depth transcriptomic characterization. In 2025, Huang et al. reported a novel protocol for generating testicular organoids from hiPSCs (Figure 7E), which not only addressed these limitations through functional testing and RNA-seq analysis but also produced a more complex model featuring a blood–testis barrier (BTB) [51]. Their protocol began with stepwise differentiation of hiPSCs into intermediate mesoderm over 4 days, in alignment with Knarston et al., but additionally included AA during the first 2 days [50]. This was followed by treatment with FGF9, BMP4, and heparin until day 7, similar to Knarston et al., resulting in increased expression of bipotential gonadal and testicular markers including *WT1*, *GATA3*, and *SOX9* [50]. These results were further confirmed by IF staining, which showed GATA4+ Sertoli cells, SOX9+ bipotential gonadal cells, Leydig cells positive for STAR, and Collagen IV+ cells, suggesting a formation of epithelial basement membrane. To initiate organoid formation, the differentiated bipotential gonadal cells were dissociated and reaggregated in 3D, using a hanging-drop method, followed by culture for 3 days in medium supplemented with EGF, FSH, LH, IGF, and RA. After initial organoid formation, the testicular organoids were transferred to rotation culture and maintained until day 18. Molecular characterization revealed enrichment of bipotential gonadal markers *GATA4*, *WT1*, and *EMX2* at day 8, with expression gradually declining thereafter. In contrast, Sertoli cell markers, *SOX9* and *CLDN11*, and most excitingly, *FSHR*, showed increasing expression over time. Throughout the organoid culture period, Leydig cell markers, *CYP17A1* and *HSD3B*, exhibited consistently high and stable expression. Cross-sections of testicular organoids on day 8 showed that Sertoli cells (SOX9+) were localized at the periphery of the organoids, while bipotential gonadal cells (GATA4+) were distributed throughout the organoids, suggesting a tubule-like organization, which was morphologically confirmed on H&E staining. Leydig cells (HSD3B+) and bipotential gonadal cells were positioned medial to the Sertoli cells. Evidence for BTB formation was supported not only by the presence of Collagen IV+ basement membrane structures, but also by transmission electron microscopy, revealing tight junctions between polarized epithelial cells [51].

The transcriptome of day-8 organoids was compared to hiPSCs and adult human testicular tissue (HTT). Compared to hiPSCs, the organoids exhibited 3627 DEGs, and GO analysis revealed enrichment of pathways related to spermatogenesis and extracellular matrix organization. Compared to HTT samples there were 2772 DEGs, but the organoids displayed comparable expression levels of key testicular markers, including *GATA3*, *CLDN11*, *STAR*, and *SOX9*. Next, the authors performed scRNA-seq on the organoids and integrated the data with a human fetal testis dataset [166]. UMAP clustering revealed that the organoids consisted of distinct cell populations, including Sertoli (*SOX9*, *PRDX1*), gonadal (*GATA4*), Leydig (*INHBA*), mesenchymal (*COL1A1*, *COL1A2*), endothelial (*SLIT2*), myoid (*MYH11*) and epithelial (EPCAM, CDH1) cells. The organoids showed strong similarity to human fetal testis samples, with the highest correlation observed with 8-week fetal testis tissue. These findings indicate that the protocol developed by Huang et al. more closely represents the early stages of human testicular development, rather than adult testicular tissue [51].

To investigate whether the organoids respond to hormonal stimulation and possess steroidogenic capacity, Huang et al. examined the expression of key components of the HP–gonadal axis, including the LH, FSH, hCG, and androgen receptor. The protein levels of these receptors were comparable to those observed in human testicular tissue. Moreover, treatment of organoids with FSH resulted in elevated lactate production, a metabolite produced by Sertoli cells in response to FSH stimulation to sustain germ cell metabolism [167]. In addition, LH treatment led to increased testosterone levels in the culture medium, indicating the presence of functional, steroidogenic Leydig cells. To evaluate the functionality of the BTB, the authors employed sulfo-NHS-LC-biotin, a water-soluble, membrane-impermeant tracer. Even at high concentrations, sulfo-NHS-LC-biotin was unable to penetrate the organoids, demonstrating selective permeability consistent with a functional BTB. Overall, Huang et al. presented a rigorously characterized testicular organoid model that closely mirrors the transcriptional landscape of developing human testes and exhibits both hormone responsiveness and steroidogenic activity (Table 1) [51]. Together, these features position this organoid model as a versatile tool for advancing future studies of human testicular development and function.

## 8. Conclusions

In the last decade, there has been a boom in the development of ESC- and iPSC-based human organoid models. These new models enabled many novel research approaches for understanding human biology and pathobiology. Here we presented hESC/hiPSC-based organoid models of the endocrine axes, which have been developed to date, ranging from the hypothalamus and pituitary to their downstream target organs.

Recent advances in hiPSCs- and hESCs-based hypothalamic organoid modeling have yielded a diverse array of protocols (Figure 2B–F), each tailored to specific research goals. Qian et al. first introduced a general hiPSC-derived hypothalamus organoid model, providing the foundation for later models [26]. Huang et al. established a protocol for developing ARCOs aiming to study region-specific PWS pathology, later refined by Shen et al. to reduce organoid-to-organoid variability and cell death [29,33]. Ozaki et al. [30] focused on establishing a rostral hypothalamic organoid model enriched in AVP+ neurons to model FNDI, while Miwata et al. [34] aimed to generate neurospheres to pave the way towards hypothalamic regenerative medicine, and Sarrafha et al. [31] created a hypothalamus organoid model enriched in TH+ neurons, offering new opportunities for dopaminergic research. Furthermore, Nemoto et al. established a hypothalamus organoid model to demonstrate the capacity of epigenome editing to restore the function of PWS-related genes, thereby underscoring its potential as a novel therapeutic tool [28]. While these models have proven valuable for studying hypothalamic structure and function during disease, future efforts must be made to expand modeling to encompass the entire HP axis. Remarkably, OXT+ and AVP+ neurons, which are connected to the posterior pituitary in vivo, are present in several models [27,30,31]. Furthermore, TRH+ cells have been observed in certain models as well [30,31]. However, deep scrutiny regarding the presence, functionality, and maturity of the hormone-producing cell types that play a central role in communicating with the pituitary is necessary. Additionally, given the limited availability of protocols targeting specification of these populations, including CRH+, TRH+, GnRH+ and GHRH+ neurons, there remains substantial room for innovation (Table 1).

HP organoids exhibit striking potential to model HP axis dynamics and to facilitate investigation of human pituitary biology (Figure 3B–D). Several HP organoid models demonstrate ACTH production and provide evidence of hormonal responsiveness [35,36,38,39]. Furthermore, Kasai et al. demonstrated functional intercompartmental communication between CRH+ hypothalamic neurons and corticotropes, an initial step towards establishing hypothalamus–corticotrope communication (Table 1) [36]. In the HP organoid model of Ozone et al., GH emerges as the second most abundant pituitary hormone, suggesting a potential for hypothalamus–somatotropic interaction, which remains to be validated [35]. Kasai et al. reported the presence of GHRH+ neurons in HP organoids; nevertheless, information on GH expression was not provided [36]. Regardless of its presence, integration with GH-producing models, such as the model by Ozone et al., may offer an avenue towards modeling hypothalamus–somatotropic communication. Interestingly, current HP organoid models show only limited TSH expression, with TSH+ cells comprising less than 1% [35,38,39]. Increasing the number of TSH+ cells could allow integration with hypothalamic models containing TRH+ neurons [30,31,36], although these neurons are scarce and unquantified. A similar limitation is observed for gonadotropes, which are present only at minimal levels [35,38,39], and attempts to increase their numbers were unsuccessful [168]. Moreover, none of the HP models reported GnRH+ neurons, indicating that the establishment of functional hypothalamus–gonadotrope communication still requires further investigation. Furthermore, none of the studies compared the transcriptomic profiles of the organoids to adult or fetal primary tissue, indicating that the level of maturity of these organoids remains unclear and requires further investigation. Altogether, current HP organoid models support initial exploration of interactions between the hypothalamus and somatotropes or corticotropes. Nevertheless, their capacity to secrete GH and ACTH, as well as the presence of corresponding regulatory neural types, open the doors to model the entire endocrine axis by incorporating downstream target organs (e.g., the liver and adrenal gland, respectively).

Current thyroid organoid protocols offer a physiologically relevant platform to study human thyroid biology (Table 1) (Figure 4B–D). Thyroid organoids developed by Romitti et al. [40], Undeutsch et al. [42], and Venegas et al. [43] show expression of *TSHR* and can respond to TSH stimulation, rendering them suitable to model the pituitary–thyroid axis. Moreover, Romitti et al. identified mature thyrocytes with transcriptomic profiles closely matching those of adult thyroid tissue, suggesting that these organoids can be used to study both developmental and adult thyroid biology [40]. Undeutsch et al. claim that thyroid organoids can be generated under serum-free conditions, making them suitable for human transplantation; however, the use of Matrigel calls this claim into question [42]. Furthermore, Nazzari et al. [41] demonstrated that organoids, generated using Romitti’s protocol, express sex hormone receptors and, importantly, respond to hormonal treatments (E2, PG, and DHT), providing new opportunities to explore thyroid response to gonadal hormones and ovarian/testicular–thyroid communication. Especially, the ovaroids developed by Smela et al. would be exciting to combine with Romitti’s thyroid organoids, as they can secrete E2 and PG [40,47]. Finally, the T4 production capacity observed in organoids developed by Venegas et al. [43] and Romitti et al. [40] offers a valuable framework to investigate thyroid–peripheral organ crosstalk, such as the thyroid–liver axis [169]. This could be further advanced by integrating the different organoids using organ-on-chip technology, which is already being developed for mESC-derived thyroid organoids [170].

Protocols for the development of human adrenal cortex organoids remain limited (Table 1) (Figure 5B,C). Sasaki et al. produced two protocols to develop hiPSC-based adrenal organoids that closely resemble in vivo conditions at the transcriptomic level, contain key cell types that are essential in the adrenal cortex, are capable of generating adrenal cortex steroids, whose levels can be regulated by ACTH, and provide a useful platform for investigating novel pathways involved in adrenal cortex development [44,45]. Their responsiveness to ACTH makes them a great fit with any HP organoid model to investigate HP–adrenal gland axis biology. However, these adrenal gland organoids still require more extensive characterization to determine how closely they recapitulate the in vivo adrenal gland. Although the xenografted organoids showed high transcriptomic correlation with human adult samples, the maturation status of not-xenografted organoids and their morphological resemblance to the adult adrenal gland need to be explored further [45]. Moreover, the organoids appear to lack an inner medulla, an essential component of the adrenal cortex. The presence of chromaffin cells and neural cell types must be evaluated to determine the extent to which an inner medulla is represented within the organoids. Overall, these organoids remain a highly promising tool, not only for unraveling communication dynamics within the HP–adrenal gland axis, but also to investigate interactions with peripheral organs.

The development of ovarian organoids has posed a significant challenge, as most induction protocols have primarily yielded PGCLCs, while robust differentiation protocols for GCs were unavailable. Yu et al. addressed this limitation by aggregating iPSC-derived PGCLCs with primary GCs, generating structures that exhibited folliculogenic capacity following xenotransplantation into mice (Table 1) (Figure 6B) [46]. However, the functional competence of these aggregates in terms of steroidogenesis requires further investigation. Moreover, their dependence on xenotransplantation to initiate folliculogenesis introduces substantial experimental constraints, limiting their applicability for studies of the HP–gonadal axis. In contrast, the ovaroids, developed by Smela et al., demonstrate responsiveness to FSH stimulation and can produce E2 and PG, highlighting their potential utility in HP–gonadal axis research (Table 1) (Figure 6C) [47]. Transcriptomic projection of the organoids onto a human fetal ovary reference atlas revealed strong overlap, suggesting that the model more closely recapitulates an embryonic state rather than adult tissue [47]. Current ovaroid models lack intrinsic self-organizing capacity and still rely on the aggregation of two distinct cell populations, rendering them a labor-intensive model that requires careful experimental coordination.

The last decade has been fruitful for the development of novel human testicular organoids from pluripotent stem cells, resulting in five notable protocols (Table 1) (Figure 7C–E). The approaches developed by Pryzhkova and Jordan [48], as well as Robinson et al. [49], generate complex tubular-like structures, which partially recapitulate testicular organization, but they lack comprehensive characterization and functional validation. In contrast, the organoids developed by Knarston et al. [50] are well characterized and exhibit signs of steroidogenic capacity. These organoids may be valuable for studies aimed at uncovering regulatory pathways governing Sertoli, Leydig, and bipotential gonadal cell populations. However, their functional competence must be further validated before they can be applied to studies of HP–gonad interactions. Among the available models, the organoids, established by Huang et al. [51], represent a further advance, as they are extensively characterized at both the transcriptomic and protein levels. Importantly, these organoids respond to FSH and LH stimulation and can produce testosterone, making them a promising platform for integration with HP–organoids on chip. Nevertheless, their capacity to support spermatogenesis, particularly the generation of spermatids or spermatozoa, remains to be demonstrated and may require further optimization. Moreover, these organoids more closely recapitulate early stages of human testicular development, and their potential to study adult testis physiology will likely require additional maturation toward later developmental stages.

Despite the rapid progress in PSC-derived models of HP–endocrine axes, several intrinsic limitations of PSC-based organoid systems remain. Differentiation protocols are typically long, labor-intensive, and costly, requiring prolonged culture periods, high reagent consumption, and tightly controlled handling. In addition, variability in PSC lines, including differences in differentiation propensity, epigenetic status, and donor dependent transcriptional profiles, introduces substantial batch-to-batch and line-to-line heterogeneity [18,107]. Genomic instability, which may arise during long-term passaging or reprogramming, further compromises reproducibility and may bias lineage specification [18,171]. Moreover, although many protocols successfully generate early developmental cell states, most PSC-derived organoids still exhibit limited maturation, often resembling fetal rather than adult tissue, as, for example, Huang et al. observed in their testis organoid model [51]. This immaturity affects hormone production, receptor expression, and responsiveness to physiological cues, thereby constraining their applicability for modeling long-term endocrine function [172]. Since PSC-derived organoids typically only reach fetal-like maturity, they are naturally powerful tools for studying human development [173]. Importantly, this embryonic or fetal-like identity also makes PSC-derived organoids particularly valuable for modeling developmental and congenital diseases, such as PWS and congenital pituitary hypoplasia, in which early hypothalamic and pituitary defects are central to pathogenesis [28,33,76]. In contrast, adult-stem-cell-derived organoids usually capture adult physiological states more faithfully and are therefore widely used in disease modeling [174]. However, for many endocrine tissues, including the hypothalamus, adrenal cortex, and early gonadal lineages, adult-stem-cell-based approaches are not available, making PSC-derived organoids indispensable even for disease focused studies.

The interplay between the hypothalamus, pituitary and target organs/glands is a complex biological system that requires coordinated interactions among these distinct organ systems, operating differently in women and men. This level of complexity is challenging to recapitulate in vitro; however, recent advances in organoid technologies provide valuable tools to study individual components of this system. Integrating multiple PSC-derived organoids into a unified organ-on-chip platform poses additional challenges. First, coculturing distinct organoids requires precise synchronization of their developmental timing, yet most protocols differ markedly in duration, patterning requirements, and maturation trajectories [175]. As a result, combining tissue types at compatible developmental stages is technically difficult. Second, each organoid type often depends on specific media compositions, growth factors, oxygen levels, and mechanical conditions; identifying a common culture environment that supports all tissues without compromising function remains a major barrier [176]. Third, long-term maintenance on chip is hindered by organoid fragility and limited lifespan, as well as the need for stable perfusion and microfluidic control to prevent shear-stress-induced damage [177]. Additionally, achieving functional physiological coupling, such as establishing directional hormone gradients, vascular-like perfusion, or neuroendocrine connectivity, requires microengineering solutions that are not yet widely accessible. Finally, current microfluidic devices and perfusion systems remain costly and technically demanding to operate, making organ-on-chip experiments economically prohibitive for many laboratories [178]. Overcoming these biological, technical, and economic hurdles will be essential to fully exploit PSC-based organoids in future endocrine-on-chip applications.

With this review, we aimed to give an overview of existing hESC- and hiPSC-based organoid models of the complex endocrine network. We highlighted the models’ respective strengths and limitations in covering human endocrinology, as well as proposing potential multi-organoid combinations based on their functional capacities. Although a diverse array of organoids is now available to address organ-specific questions, many aspects of inter-organ communication and functional integration remain unresolved. With advances in organ-on-chip technologies, many of these questions may be addressed in what could represent a further rise in organoid biology.

## Figures and Tables

**Table 1:** Summary of existing organoid models and their characteristics (from which cell source they were generated, what cell types are (not) present in the organoids, and whether they show endocrine functionality). We used the cell type nomenclature as reported in the respective studies. Cell types were classified as present when marker expression was confirmed at both the transcriptomic and protein levels. If a cell type was detected only at the transcriptomic level, it was indicated with an asterisk (*). If a cell type was not reported in the study, it was considered as a missing cell type. Endocrine functionality was defined as the ability of organoids to secrete hormones or respond to hormonal stimulation. Studies were classified as ‘Yes’ if they explored and demonstrated hormonal secretion or responsiveness. Studies were classified as ‘Unknown’ if these capabilities were not investigated. If endocrine function was not detected in organoids, then the study was classified as ‘No’. Given the complexity of the hypothalamus and its diverse neuronal subtypes, all remaining neurons are grouped under ‘other hypothalamic neurons’.

Protocol	Cell Source	Present Cell Types (Transcriptomically Defined *)	Missing Cell Types	Endocrine Functionality
Hypothalamus Organoids				
Qian et al. 2018 [26]	hiPSCs	Peptidergic neurons Neuroendocrine neurons	All other neurons	Unknown
Huang et al. 2021 [33]	hiPSCs	Peptidergic neurons GABAergic neuron Neural progenitors * Intermediate progenitor cells * Neuroendocrine neurons * Peptidergic neurons *	All other neurons	Unknown
Ozaki et al. 2022 [30]	hiPSCs	Peptidergic neurons Neuroendocrine neurons Excitatory peptidergic neurons	All other neurons	Unknown
Sarrafha et al. 2023 [31]	hESCs hiPSCs	Dopaminergic neurons Peptidergic neurons Neuroendocrine neurons Astrocytes Glutamatergic neurons GABAergic neurons * Preoptic area neurons * Midbrain neurons * Neuroendocrine-like cells * Intermediate progenitor cells * Radial glial cells * Oligodendrocytes * Ependymal cells * Tanycytes * Endothelial cells * Microglia * Vascular and leptomeningeal cells * Oligodendrocyte precursor cells *	All other neurons	Unknown
Miwata et al. 2023 [34]	hESCs	Peptidergic neurons	All other neurons	Unknown
Shen et al. 2025 [29]	hiPSCs	Peptidergic neurons Dopaminergic neurons	All other neurons	Unknown
Nemoto et al. 2025 [28]	hiPSCs	Hypothalamic neurons * Preoptic telencephalon neurons * Cycling cells * Hypothalamic progenitors *	All other neurons	Unknown
Pituitary Organoids				
Ozone et al. 2016 [35]	hESCs	Corticotropes Somatotropes Thyrotropes Lactotropes Gonadotropes	Pituitary stem cells Folliculostellate cells	Yes
Kasai et al. 2020 [36] and follow-up study by Miyake et al. 2022 [37]	hiPSCs	Neuroendocrine neurons Peptidergic neurons Corticotropes Lactotropes	Pituitary stem cells Folliculostellate cells Somatotropes Thyrotropes Gonadotropes	Yes
Taga et al. 2023 [38]	hESCs hiPSCs	Corticotropes Somatotropes Thyrotropes Lactotropes Gonadotropes Pituitary stem cells	Folliculostellate cells	Yes
Wang et al. 2025 [39]	hiPSCs	Corticotropes Lactotropes Somatotropes * Thyrotropes * Gonadotropes * Glutamatergic neurons * GABAergic neurons * Dopaminergic neurons * Histaminergic neurons * Cholinergic neurons * Neural stem and progenitor cells * Fibroblasts * Posterior pituicytes * Cycling cells * Ependymocytes *	Pituitary stem cells Folliculostellate cells	Yes
Thyroid Gland Organoids				
Romitti et al. 2022 [40]	hESCs	Thyroid progenitors Thyrocytes Immune cells * Neurons *	Parafollicular cells	Yes
Nazzari et al. 2024 [41]	hESCs	Thyroid progenitors Thyrocytes Immune cells * Neurons *	Parafollicular cells	Yes
Undeutsch et al. 2024 [42]	hESCs hiPSCs	Thyroid progenitors Thyrocytes Immune cells * Neurons *	Parafollicular cells	No
Venegas et al. 2025 [43]	hiPSCs	Thyroid progenitors Thyrocytes	Parafollicular cells Immune cells Neurons	Yes
Adrenal Gland Organoids				
Sakata et al. 2022 [44]	hiPSCs	Adrenal-primordium-like cells Fetal-zone-like cells Capsule-like cells *	Adult adrenal gland cell types Definitive zone cells Transitional zone cells Inner medulla cell types	Yes
Mayama et al. 2025 [45]	hiPSCs	Adrenal-primordium-like cells Definitive-zone-like cells Transitional-zone-like cells Capsule-like cells Fetal-zone-like cells	Adult adrenal gland cell types Inner medulla cell types	Yes
Ovary Organoids				
Yu et al. 2021 [46]	hESCs hiPSCs	Primordial-germ-cell-like cells Adult granulosa cells	Oocytes Theca cells Stromal cells Surface epithelium	Unknown
Smela et al. 2023 [47]	hESCs hiPSCs	Primordial-germ-cell-like cells Granulosa-like cells Stromal cells * Surface epithelium *	Oocytes Theca cells	Yes
Testis Organoids				
Pryzhkova and Jordan 2020 [48]	hESCs	Pre-Sertoli cells Gonadal progenitors Adult testicular cells	Spermatogenic cells Spermatozoa Leydig cells Endothelial cells Peritubular myoid cells Spermatogonial stem cells Epithelial cells	Unknown
Robinson et al. 2021 [49]	hiPSCs	Spermatogonial stem cells Sertoli cells Leydig cells Endothelial cells Peritubular myoid cells	Spermatogenic cells Spermatozoa Epithelial cells	Unknown
Knarston et al. 2020 [50]	hiPSCs	Bipotential gonadal cells Sertoli cells Leydig cells	Spermatogonial stem cells Spermatogenic cells Spermatozoa Endothelial cells Peritubular myoid cells Mesenchymal cells Epithelial cells	Unknown
Huang et al. 2025 [51]	hiPSCs	Bipotential gonadal cells Sertoli cells Leydig cells Epithelial Cells Endothelial cells * Peritubular myoid cells * Mesenchymal cells *	Spermatogonial stem cells Spermatogenic cells Spermatozoa	Yes

## Data Availability

Not applicable.

## Abbreviations

The following abbreviations are used in this manuscript:

AA	Activin A
ACTH	Adrenocorticotropic hormone
ACTHD	ACTH deficiency
AMH	Anti-Müllerian hormone
ARCO	Arcuate nucleus organoids
BBT	Blood–testis barrier
BaP	Benzo[a]pyrene
CRH	Corticotropin-releasing hormone
DHEA	Dehydroepiandrosterone
DHEA-S	DHEA-sulfate
DHT	Dihydrotestosterone
DOX	Doxycycline
DX	Dexamethasone
DZ	Definitive zone
DZLC	Definitive-zone-like cell
EB	Embryoid body
EDC	Endocrine-disturbing chemicals
ESC	Embryonic stem cell
E2	β-estradiol
FACS	Fluorescence-activated cell sorting
Ff	Feeder-free
FNDI	Familial neurohypophyseal diabetes insipidus
FSH	Follicle-stimulating hormone
FZ	Fetal zone
FZLC	Fetal-zone-like cell
GC	Granulosa cells
GLC	Granulosa-like cell
GH	Growth hormone
GHRH	Growth-hormone-releasing hormone
GnRH	Gonadotropin-releasing hormone
hESC	Human embryonic stem cell
hiPSC	Human-induced pluripotent stem cell
HP	Hypothalamus–pituitary
HTT	Human testicular tissue
iPSC	Induced pluripotent stem cell
KSR	Knock-out serum replacement
LH	Luteinizing hormone
NPC	Neural progenitor cell
NPY	Neuropeptide Y
OXT	Oxytocin
PCS	Pluripotent stem cell
PG	Progesterone
PGC	Primordial germ cells
PGCLC	Primordial-germ-cell-like cell
PMN	Purmorphamine
POMC	Pro-opiomelanocortin
PRL	Prolactin
PWS	Prader–Willi syndrome
RA	Retinoic acid
RAI	Radioactive iodine
sc	Single-cell
SCID	Severe combined immunodeficient
SFEBq	Serum-free culturing of EB-like aggregates with quick reaggregation
SHH	Sonic hedgehog
snRNA-seq	Single-nucleus RNA sequencing
SSC	Spermatogonial stem cell
TF	Transcription factor
TFC	Thyroid follicular cell
TG	Thyroglobulin
TG-I	Iodinated thyroglobulin
TRH	Thyrotropin-releasing hormone
TSH	Thyroid-stimulating hormone
TZLC	Transitional-zone-like cells
T3	Triiodothyronine
T4	Thyroxine
VIP	Vasoactive intestinal peptide
zF	*Zona fasciculata*
zG	*Zona glomerulosa*
zR	*Zona reticularis*
